# Mitigating the negative impacts of tall wind turbines on bats: Vertical activity profiles and relationships to wind speed

**DOI:** 10.1371/journal.pone.0192493

**Published:** 2018-03-21

**Authors:** Sascha D. Wellig, Sébastien Nusslé, Daniela Miltner, Oliver Kohle, Olivier Glaizot, Veronika Braunisch, Martin K. Obrist, Raphaël Arlettaz

**Affiliations:** 1 Division of Conservation Biology, Institute of Ecology and Evolution, University of Bern, Erlachstrasse 9a, Bern, Switzerland; 2 KohleNusbaumer SA, Chemin de Mornex 6, Lausanne, Switzerland; 3 Museum of Zoology, Place de la Riponne 6, Lausanne, Switzerland; 4 Forest Research Institute of Baden-Württemberg, Wonnhaldestrasse 4, Freiburg, Germany; 5 WSL, Swiss Federal Institute for Forest, Snow and Landscape Research, Biodiversity and Conservation Biology, Zürcherstrasse 111, Birmensdorf, Switzerland; Centro de Investigacion Cientifica y de Educacion Superior de Ensenada Division de Fisica Aplicada, MEXICO

## Abstract

Wind turbines represent a source of hazard for bats, especially through collision with rotor blades. With increasing technical development, tall turbines (rotor-swept zone 50–150 m above ground level) are becoming widespread, yet we lack quantitative information about species active at these heights, which impedes proposing targeted mitigation recommendations for bat-friendly turbine operation. We investigated vertical activity profiles of a bat assemblage, and their relationships to wind speed, within a major valley of the European Alps where tall wind turbines are being deployed. To monitor bat activity we installed automatic recorders at sequentially increasing heights from ground level up to 65 m, with the goal to determine species-specific vertical activity profiles and to link them to wind speed. Bat call sequences were analysed with an automatic algorithm, paying particular attention to mouse-eared bats (*Myotis myotis and Myotis blythii*) and the European free-tailed bat (*Tadarida teniotis*), three locally rare species. The most often recorded bats were the Common pipistrelle (*Pipistrellus pipistrellus*) and Savi’s pipistrelle (*Hypsugo savii*). Mouse-eared bats were rarely recorded, and mostly just above ground, appearing out of risk of collision. *T*. *teniotis* had a more evenly distributed vertical activity profile, often being active at rotor level, but its activity at that height ceased above 5 ms^-1^ wind speed. Overall bat activity in the rotor-swept zone declined with increasing wind speed, dropping below 5% above 5.4 ms^-1^. Collision risk could be drastically reduced if nocturnal operation of tall wind turbines would be restricted to wind speeds above 5 ms^-1^. Such measure should be implemented year-round because *T*. *teniotis* remains active in winter. This operational restriction is likely to cause only small energy production losses at these tall wind turbines, although further analyses are needed to assess these losses precisely.

## Introduction

The continuous supply of fossil energy sources such as petrol is compromised in the long run while the global warming crisis calls for a reduction of carbon and other greenhouse gas emissions. Many nations worldwide have therefore started to seek new ways of generating more sustainable energy sources for the future, for example wind energy. At a first glance, wind energy appears to offer a perfect neutral solution for the environment, but it also has its drawbacks [1]. Conservationists have raised concerns about the impact of wind turbines on wildlife, especially upon flying vertebrates such as birds and bats [2,3]. Research about bird and bat fatalities at wind farms has established that turbines might be even more detrimental to bats than to birds [4,5]. This is largely due to the fact that most bats have a very slow life history strategy [6,7]. Any additional source of mortality might thus tremendously impact bat population dynamics, especially when populations are small in size [8]. Several studies have reported very high bat fatality rates at wind turbines [9,10,11,12], putting some local bat populations at risk of extinction while potentially also affecting distant populations in migratory species [13]. Bats are injured or killed directly when struck by turbine blades, or indirectly by decompression near blades (barotrauma, [14,15]), although the latter is still controversial [16]. There are several non-mutually exclusive explanations why bats collide with wind turbines [17]. First, they may fail to detect the rapidly approaching blades because of the extremely high rotor speed (up to 300 km h^-1^ at blade apex), in part due to the very focal character of bat sonar. Second, they may underestimate blade velocity (about ten times their flight speed) when maneuvering in the rotor-swept zone, failing to avoid collision [9]. Third, they might be attracted to wind turbine towers as such tall elements dominating the landscape might attract insects [18] or be perceived as potential roosts or even as vantage mating sites [19].

Today, modern wind turbine towers are much taller than in the past [20], which might augment negative effects on bat populations. Indeed, some studies have found a positive exponential relationship between number of bats killed and turbine tower height, whereas the size of the rotor-swept zone (diameter of rotor blades) had no influence [21,22]. On the other hand, increasing turbine heights might shift the species-specific risks towards high-height aerial hunting and commuting species while alleviating the risk for species mainly flying above ground level. In addition, species-specific risk may be modulated by environmental and wind conditions. Yet, the lack of quantitative information about species-specific vertical activity profiles impedes recommending targeted mitigation strategies.

We investigated vertical bat activity profiles in relation to wind speed at low altitude (valley bottom) in a windy stretch of the Rhône valley in the European Alps. We put a particular emphasis on three locally rare bat species: the two sibling mouse-eared bats (*Myotis myotis and M*. *blythii*) and the European free-tailed bat (*Tadarida teniotis*). Our main objective was to propose targeted evidence-based management measures for reducing the number of bat fatalities at the tall wind power plants (rotor-swept zone 50–150 m above ground) that are currently under rapid deployment.

## Materials and methods

### Ethics statement

Species data were obtained by means of automatic recording outside protected areas, therefore no permits for handling of endangered species or for access to protected areas was required. Permits for crane installation on public grounds were issued by the two concerned communities Fully and Ardon.

### Study area

The study was conducted in the Lower Rhône valley (Valais, SW Switzerland) in an area where a wind park is under development, with a first turbine installed in 2013 (model Enercon E-101 with nacelle height at 99 m above the ground, and rotor diameter of 101 m). The valley bottom is situated at approximately 500 m a.s.l., while mountain ranges culminating at more than 4000 m flank the valley in the North and South. Windmills are being installed on the Rhône valley floor, which mostly consists of high-intensity agriculture (fruit tree plantations, cropland and scattered grasslands) and settlements.

Altogether, 27 bat species have been recorded in Valais [23], with two species of high conservation concern breeding within 8 km of the planned wind farm. The European free-tailed bat (*T*. *teniotis*) is a long-distance, high-altitude aerial forager with a mostly Mediterranean distribution. This species remains active in winter down to -1°C ambient temperature, foraging mostly on flying tympanate insects such as moths [24,25]. It roosts in sheer limestone cliffs adjacent to the valley bottom [26]. The lesser mouse-eared bat (*M*. *blythii*) is a substrate gleaner specialized on orthopterans captured from grass stalks. It breeds in a church attic 2 km from the closest planned wind turbine, forming a mixed colony with the less threatened, but locally rare sibling species *M*. *myotis* [27,28,29]. More information about the local bat community is given in Supporting Information (S1 File).

### Bat recordings

Vertical bat activity was recorded from near-ground level (5 m) up to 65 m above ground level (a.g.l.), using a truck-mounted crane (as described below), at two sites situated close to the planned wind farm implantation areas (Solverse and Marais d’Ardon, E: 7° 8.841’–N: 46° 7.933’ and E: 7° 15.469’–N: 46° 11.790’, respectively). In addition, bat activity at ground level (1 m) was recorded at six foreseen wind turbine sites (ValEole 1–6, coordinates see S1 Table) in order to better assess local bat assemblages and to relate bat activity to wind speed at ground level (S1 Fig).

Bat echolocation calls were automatically recorded from dusk to dawn (recording length was adjusted to night length) with Batloggers (Elekon AG, Luzern, Switzerland) equipped with an elongated wire microphone, extended rechargeable battery pack and protective box (Strongbox, Elekon AG). These recorders operate within the 10–150 kHz frequency range; the lower frequency sensitivity was important in this study because *T*. *teniotis* emits audible echolocation calls, which enables it to feed on flying tympanate insects [25]. In the automatic mode, the Batlogger constantly monitors the microphone signal. Recording is triggered for a maximum of 15 s, including 0.5 s pre- and 1 s post-trigger, as long as an entering signal reaches a pre-set sensitivity threshold. Writing of files to memory card interrupted the recording momentarily (<2 s). By producing artificial ultrasounds originating from shaking keys within a distance of approximately 10 m, microphone functionality and sensitivity was checked before any night recording session and faulty microphones were replaced when needed. Detection distance differs between bat species depending on call intensity and frequency. This relative bias could not be accounted for but remained constant throughout the study. As recordings were restricted to nights without rainfall and low wind speeds (< 11 ms^-1^, due to crane safety reasons) we assume no confounding effect of rain, wind speed or wind direction, respectively, on detection distance.

### Vertical activity profiles

In order to investigate the vertical activity profiles of bats, a truck-mounted crane (Liebherr LTM 1200–5.1) was hired (S2 Fig). In short, two rigid metal cables were spanned from the top of the crane telescopic arm down to the ground, where they were anchored in two 2.5 ton blocks of concrete. Along each cable, five Batloggers were positioned at different heights (5, 20, 35, 50 and 65 a.g.l.), resulting in two Batloggers at each height. The microphones, protected by plastic tubes placed slightly downward (approximately 30°) to avoid not forecasted rain falling on the microphone membranes, were pointing to opposite directions (NW and SE; S2 Fig) in order to increase the detection range. Two anemometers were fixed on the cables at 10 and 70 m above the ground. Bat activity was recorded during 9 nights from July to October 2011 (S1 Table).

### Activity at foreseen wind turbine sites

A total of 12 Batloggers were used at ground level for acoustic surveys of bat activity at the six foreseen wind turbine sites. The detectors were mounted on a pole 1 m above the ground, with microphones again pointing slightly downwards (approximately 30°) (S3 Fig). A pair of ground-based bat detectors was installed within 150 m distance of a projected wind turbine site, one among fruit tree plantations, another in an adjacent open field (cropland or grassland), so as to capture the bat community in the two locally dominant habitat types. Constant measures of wind speed were obtained from a nearby anemometer situated at 35 m a.g.l.. The surveys took place during 11 nights in July-October 2011 and eight nights in May-June 2012 (S1 Table). Due to absence of bat activity on October 14^th^ and 15^th^ 2011 (cold nights) and on May 16^th^ 2012 (adverse weather), we excluded these recording nights from our analyses.

### Data analysis

#### Data extraction and bat identification

The software BatScope 2.0 was used to attribute the recorded bat echolocation call sequences to species or groups of species, based on a reference database containing 20,000 calls from 27 species (S2 File). As a recorded sequence corresponds to a bat passing next to the microphone, we shall term it bat pass hereafter. Only bat calls with a signal-to-noise ratio (SNR) greater than 30 dB were retained. For each continuous sequence, single calls were cut out, parameterised and subsequently identified using the BatScope 2.0 automatic classification algorithms, which attribute a probability of species identity to each single call. Finally, the call information was summarized over a sequence for final species assignment. Sequences with a probability of correct classification lower than 80% were discarded. We had to pool the recordings of the two mouse-eared bat species *M*. *myotis* and *M*. *blythii* because their calls are almost impossible to separate [30]. For those two species and *T*. *teniotis*, we additionally checked all sequences visually in order to avoid too conservative out-filtering.

#### Quantifying the number of bat passes

The numbers of bat passes per hour recorded by the two devices installed at each height along the cables were averaged in order to retain only one activity index per height. This was necessary given that ca 30% of bat passes were recorded simultaneously by the two detectors situated at the same height (a recording “overlap” was assumed if trigger times of recordings from two different devices fell within 1 s) (S4 Fig). Although there was a less pronounced overlap in recordings between two adjacent heights due to longer distance in between (S4 Fig), we did not account for this potential bias because it was not possible to allocate bat passes recorded simultaneously to a single height while averaging between height levels would have made no sense. This resulted in an overall overestimation of bat pass counts, which we considered as evenly distributed across height levels and thus as affecting neither the statistical outcome nor the interpretation of vertical activity patterns. To build vertical activity profiles, we used the absolute number of bat passes per hour at each height, considering 1) all recorded species pooled; 2) the two most common recorded species (*P*. *pipistrellus* and *H*. *savii*); 3) the rare target species (*M*. *myotis/M*. *blythii* and *T*. *teniotis*). Bat activity at ground level at all foreseen wind turbine sites was also estimated from the number of bat passes per hour.

#### Effect of wind speed on bat activity

Wind speed for each height at the crane was interpolated from the data recorded with the anemometers at 10 and 70 m a.g.l. (S2 Table) using Eq (1).

$$v_{x}=v_{10m}+\left(v_{70m}-v_{10m}\right)\;*\;ln\left(h_{x}/10\right)/ln\left(h_{70m}/10\right)$$ (1)

where *v*_*x*_ is the average wind speed per hour [ms^-1^]; *v*_*10m*_ and *v*_*70m*_ are hourly averages of wind speed obtained from anemometers on the crane at 10 and 70 m a.g.l., respectively; *h*_*70m*_ is height 70 m a.g.l. and *h*_*x*_ is the height (5–65 m a.g.l.) for which the wind speed was calculated. For the six foreseen wind turbine sites, we extrapolated wind speed at 5 m a.g.l. from measures obtained at 35 m a.g.l., using Eq (2),

$$v_{5m}=v_{35m}\;*\;(ln(h_{5m})/0.003)/(ln(h_{35m})/0.003)$$ (2)

where *v*_*35m*_ is the hourly average of wind speed [ms^-1^] at 35 m a.g.l.; *h*_*35m*_ is height 35 m a.g.l. and *h*_*5m*_ is the height 5 m a.g.l..

Eqs (1) and (2) are related to the logarithmic wind profile, which is commonly used to extrapolate wind speed from different heights [31]. Both formulas were evaluated with regard to their actual fit to local wind speed conditions, which had been described mathematically by local wind specialists (KohleNusbaumer SA) (Pearson’s product-moment correlation; t = 7470.7, df = 11, p < 0.001 and t = 7706.3, df = 11, p < 0.001, respectively; S5 Fig).

In order to appraise the relationships between bat activity and wind speed, we relied on three different metrics. We first calculated a *probability of activity occurrence per hour (p)* using logistic regression. For this, we transformed the raw activity data (number of bat passes per hour) into simple presence/absence (binomial distribution) per hour. To avoid overrating single bat passing events of common and abundant species, we considered, by convention, that activity corresponded to more than one bat pass per hour, while absence of activity corresponded to one or zero bat pass per hour. However, for the three rare species (*M*. *myotis/M*. *blythii* and *T*. *teniotis)*, due to data scarcity, activity was conservatively defined as >0 bat pass per hour. Given the much smaller proportion of hours with total absence for the most common detected bat species this also resulted in a more balanced dataset for logistic modelling. The probability of activity occurrence per hour as a function of wind speed was then modelled according to Eq (3):

p=exp(α*X+β)/(1+exp(α*X+β)) (3)

with *α* being the slope and *β* the intercept of the modeled relationships between bat activity and wind speed in metric 1 (S3 Table), and *X* being 1-hourly wind speed (absolute values from 0 to 9 ms^-1^).

Secondly, we calculated a *projected activity occurrence per hour (q)*, which was defined according to Eq (4) as the product of the mean number of bat passes per hour occurring at a given wind speed (1-hourly values rounded to absolute values from 0–9 ms^-1^) *N*_*(X)*,_ multiplied by the probability of activity occurrence per hour *p*.

$$q=p*N_{\left(X\right)}$$ (4)

This second metric was used to avoid either the possible bias incurred by having the same individual bat recurrently foraging around the recorder (inherent to the number of bat passes per hour) and the extremely conservative approach which consists in merely considering the probability of occurrence of activity per hour above. In other words, this projected activity occurrence per hour is likely to better reflect real bat activity. Information obtained at the crane (5–65 m height) and at ground level was used in conjunction to investigate the relationships between bat activity and wind speed, with respect to height.

As a third metric, we used the *cumulative number of bat passes per hour* with respect to wind speed to further identify which wind speed thresholds induced a tangible reduction of activity (here set at 95%) in the different species.

### Statistical analysis

For the statistical analysis of vertical activity profiles (5–65 m a.g.l.) we relied on likelihood ratio tests, which compared full models with null models. Full models were Generalized Linear Mixed Models (GLMMs) describing number of bat passes per hour as a function of height (as linear and quadratic term), with recording night as a random factor. The corresponding null model excluded the variable height. This was done for all species pooled, then separately for each of the two most commonly recorded bat species (*P*. *pipistrellus* and *H*. *savii*) as well as for the three rare species (*M*. *myotis/M*. *blythii* and *T*. *teniotis*).

To characterise species-specific activity profiles, in particular to distinguish patterns of the high aerial forager *T*. *teniotis* from middle height foragers (*P*. *pipistrellus* and *H*. *savii*), we calculated GLMMs describing the probability of activity occurrence per recording night as a function of the interaction of species and height, with recording night as a random factor. If analysis of covariance (ancova) delivered a significant effect for the interaction of species and height, we performed another species-specific ancova with height as fixed effect and recording night as a random factor.

To model vertical activity profiles up to 150 m a.g.l. (upper range of rotor-swept area) we used linear models (LMs) describing the mean number of bat passes per recording night as a function of the natural logarithm of height.

For analysing the effect of wind speed on bat activity we also used likelihood ratio tests, comparing full GLMMs of probability of activity occurrence per hour (presence/absence; first index above) as a function of wind speed (with recording night as a random factor) with null models (which exclude the variable wind speed). Effects of wind speed on bat activity were assessed at ground level (at the foreseen turbine sites), for all height levels pooled, for the dangerous, rotor-swept zone only (≥ 50 m a.g.l.), as well as for the 2 height classes (pooling the data at 5 and 20 m, 50 and 65 m a.g.l.). Statistical analyses were performed with the software R 2.13.1 [32] using the library *lme4* for GLMMs.

## Results

### Species data

We collected a total of 1,754 bat passes at the truck-mounted crane during 9 nights. Because recordings from the two recorders situated at the same height were averaged, subsequent analyses were based on 877 bat passes. Eleven bat species or groups of species were identified. The two most common species were *P*. *pipistrellus* (61.3%) and *H*. *savii* (18.2%). The rare *T*. *teniotis* and *M*. *myotis*/*M*. *blythii* were rather scarce, representing 6.3% and 1.3% of all recordings, respectively (S4 Table). Ground-level recordings at the foreseen wind turbine sites yielded a total of 2,551 bat passes, attributed to 15 species or groups of species. The most often recorded species were *P*. *pipistrellus* (81.4% of passes), the *P*. *kuhlii*/*P*. *nathusii* species group (4.3%) and *H*. *savii* (3.7%). Recordings of the rare species *M*. *myotis*/*M*. *blythii* (3.8%) and *T*. *teniotis* (2.7%) were again scarce (S5 Table). At the crane and at the six foreseen wind turbine sites, bat activity showed a clear peak at dusk (S6 Fig).

### Vertical activity profiles

**Fig 1:**
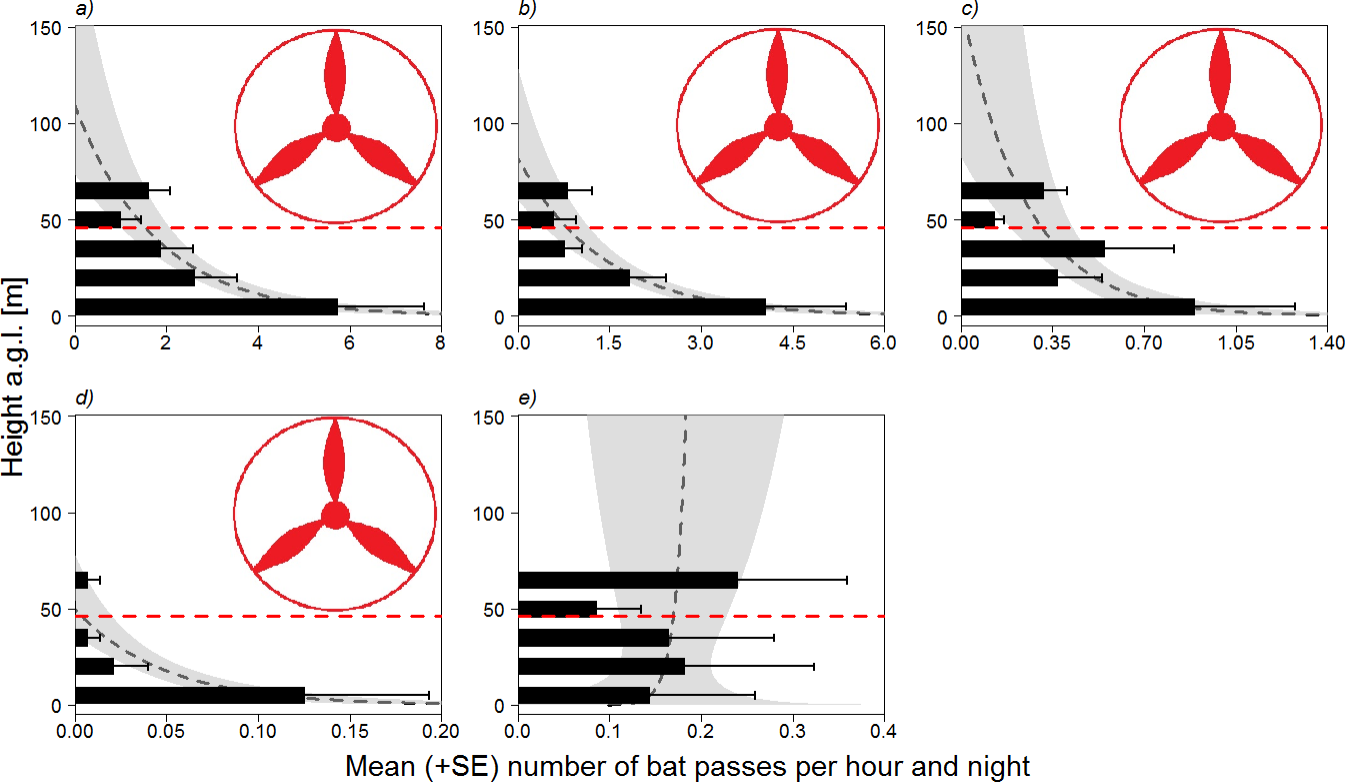
Observed (up to 65 m a.g.l.) and projected (>65 m a.g.l.) vertical bat activity profiles (nightly average + SE–the latter expressing between night variation–with 95% confidence intervals of projections shaded in grey) constructed from the hourly number of bat passes recorded at different heights. a) all species pooled, b) *Pipistrellus pipistrellus*, c) *Hypsugo savii*, d) *Myotis myotis/Myotis blythii* and e) *Tadarida teniotis*. For more realistic representation the response variable is on the X axis, height a.g.l. being the vertical (Y) axis. The red dashed line represents the lower limit (50 m) of the rotor-swept zone as depicted by the rotor icon (not represented in 1e for enhacing clarity).

The vertical distribution of activity for all species pooled, built from the hourly number of bat passes per night, shows that most activity took place at lower height (below 50 m, i.e. outside the rotor-swept zone) (Fig 1A). There was a significant difference between the model including height and the null model (likelihood-ratio test: chi-squared χ^2^ = 9.76, df = 1, p = 0.002), revealing a clear effect of height upon activity. A similar pattern was found for the most frequently recorded species *P*. *pipistrellus* and *H*. *savii*, but a significant difference against the null model was only evident for *P*. *pipistrellus* (χ^2^ = 11.31, df = 1, p < 0.001; Fig 1B and 1C). As regards the rare species, most activity of *M*. *myotis/M*. *blythii* was recorded below 20 m (Fig 1D). This model was also different from the null model (χ^2^ = 6.25, df = 1, p = 0.012). The vertical activity profile of *T*. *teniotis* is opposite to other species, increasing with height. Its profile differs from those of *M*. *myotis/M*. *blythii* (GLMM ancova with n = 90 observations from 9 different nights, z = 2.206, p = 0.027; Fig 1E) and *P*. *pipistrellus* (n = 90, z = 2.597, p = 0.009), which both decreased with height (*P*. *pipistrellus*: n = 45, z = -2.292, p = 0.022), although the relationship was only marginally significant for *M*. *myotis/M*. *blythii* (n = 45, z = -1.889, p = 0.059). A difference was also found between *T*. *teniotis* and *H*. *savii* (n = 90, z = 2.377, p = 0.018), with the activity of the latter also decreasing with height (n = 45, z = -2.048, p < 0.041).

Within the tested height range (i.e. up to 65 m a.g.l.; Fig 1), 19% of bat activity (all species pooled) took place within the potentially hazardous rotor-swept zone. There are, however, noticeable differences between species: 15% of the activity of *P*. *pipistrellus* and only 4% of the activity of *M*. *myotis/blythii* would take place at dangerous heights. In contrast, these figures reach 19% and 38% for *H*. *savii* and *T*. *teniotis*, respectively. An extrapolation of the vertical activity profiles above 65 m and up to 150 m (entire rotor-swept zone)–thereby assuming a continuation of the curvilinear profiles from 65 to 150 m a.g.l. (Fig 1)–suggests that as much as 23% of overall bat activity would take place in the risky zone (*P*. *pipistrellus*: 12%; *H*. *savii*: 38%; *M*. *myotis/M*. *blythii*: no modeled activity above 50 m; *T*. *teniotis*: 71%).

### Effects of wind speed on bat activity

**Fig 2:**
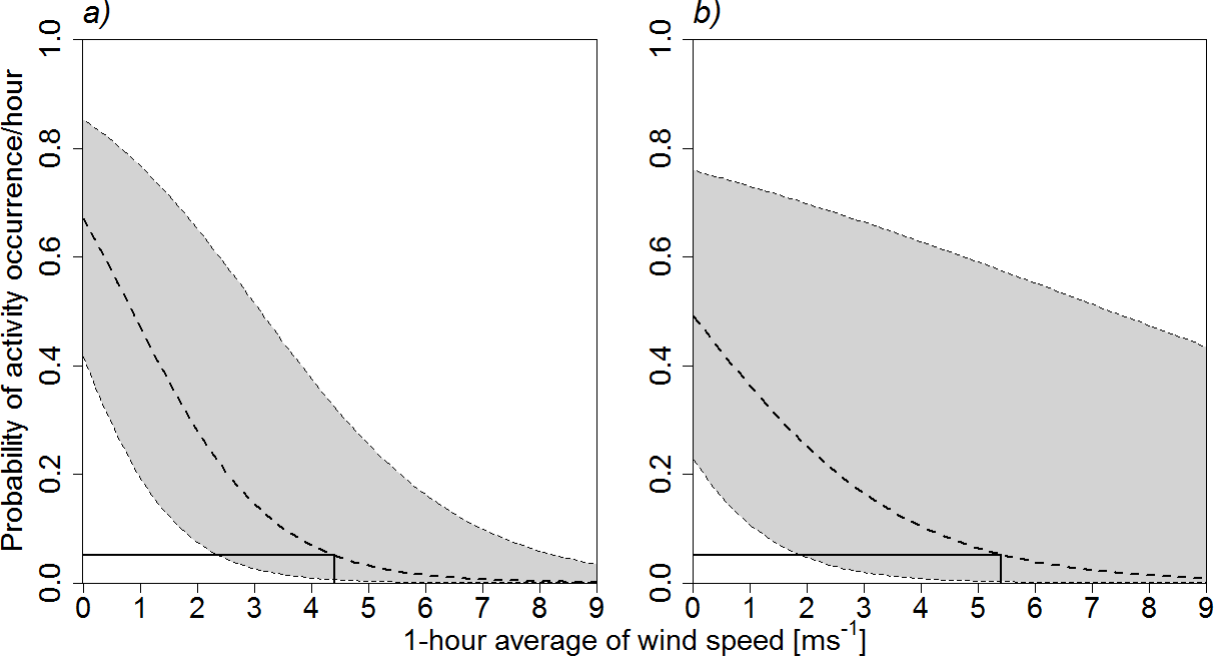
Probability of hourly bat activity occurrence in relation to mean hourly wind speed (mean ± 95% confidence intervals) at the truck-mounted crane for all species pooled. a) all heights; b) heights > 50 m a.g.l., i.e. in the dangerous rotor-swept zone. The black solid lines indicate the wind speed at which the probability of activity occurrence drops below 5%.

The probability of activity occurrence per hour (metric 1) for all species pooled significantly decreased with increasing wind speed at the truck-mounted crane. The full model was different from the null model (χ^2^ = 37.53, df = 1, p < 0.001; Fig 2A). Above a wind speed of 4.4 ms^-1^, the probability of activity occurrence per hour dropped below 5% when pooling data from all heights; this threshold was 5.4 ms^-1^ when considering only heights above 50 m, i.e. the dangerous rotor-swept zone (the latter model was also different from the null model; χ^2^ = 6.88, df = 1, p = 0.009; Fig 2B).

The projected activity occurrence per hour (metric 2) at the crane decreased with increasing wind speed, for all species pooled (S7 Fig), in a similar manner to probability of activity occurrence per hour (Fig 2). There was almost no bat activity above a wind speed of ca 5 ms^-1^; in *M*. *myotis/M*. *blythii* activity already ceased above a wind speed of 2 ms^-1^. A similar pattern was found when considering the dangerous zone only (≥ 50 m a.g.l.; S7 Fig). Except *T*. *teniotis*, all bat species became less active at the higher height for a same given wind speed.

**Fig 3:**
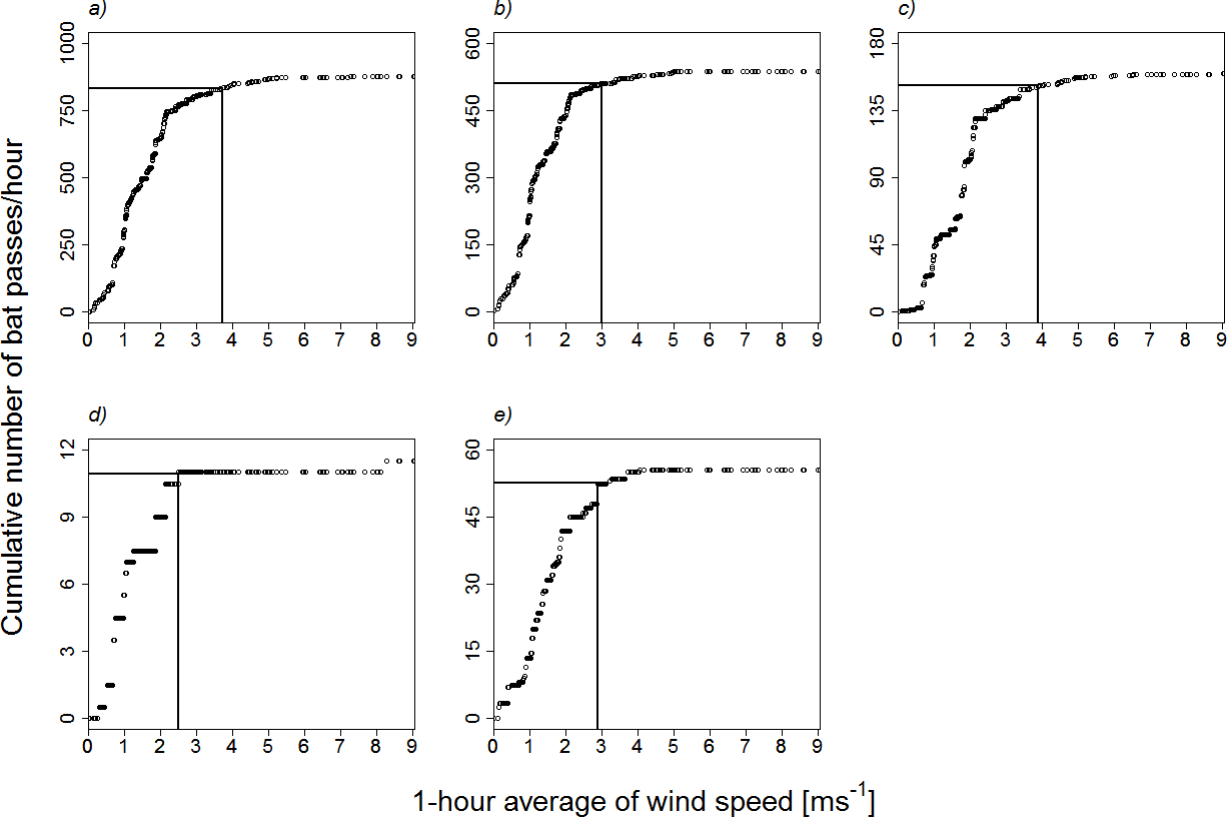
Cumulative number of bat passes per hour in relation to mean hourly wind speed at the truck-mounted crane. a) all species pooled, b) *Pipistrellus pipistrellus*, c) *Hypsugo savii*, d) *Myotis myotis/Myotis blythii* and e) *Tadarida teniotis*. The black line indicates 95% of the asymptote.

The cumulative number of bat passes per hour (metric 3) at the crane shows that 95% of the asymptote was typically reached around 2.5–3.9 ms^-1^, depending on the species, with almost no activity above approximately 4 ms^-1^ for all species (Fig 3). Note however that the thresholds are lower in *P*. *pipistrellus* and *M*. *myotis/M*. *blythii* (Fig 3B and 3D) than in the other species, which thus appear more sensitive to increases in wind speed.

**Fig 4:**
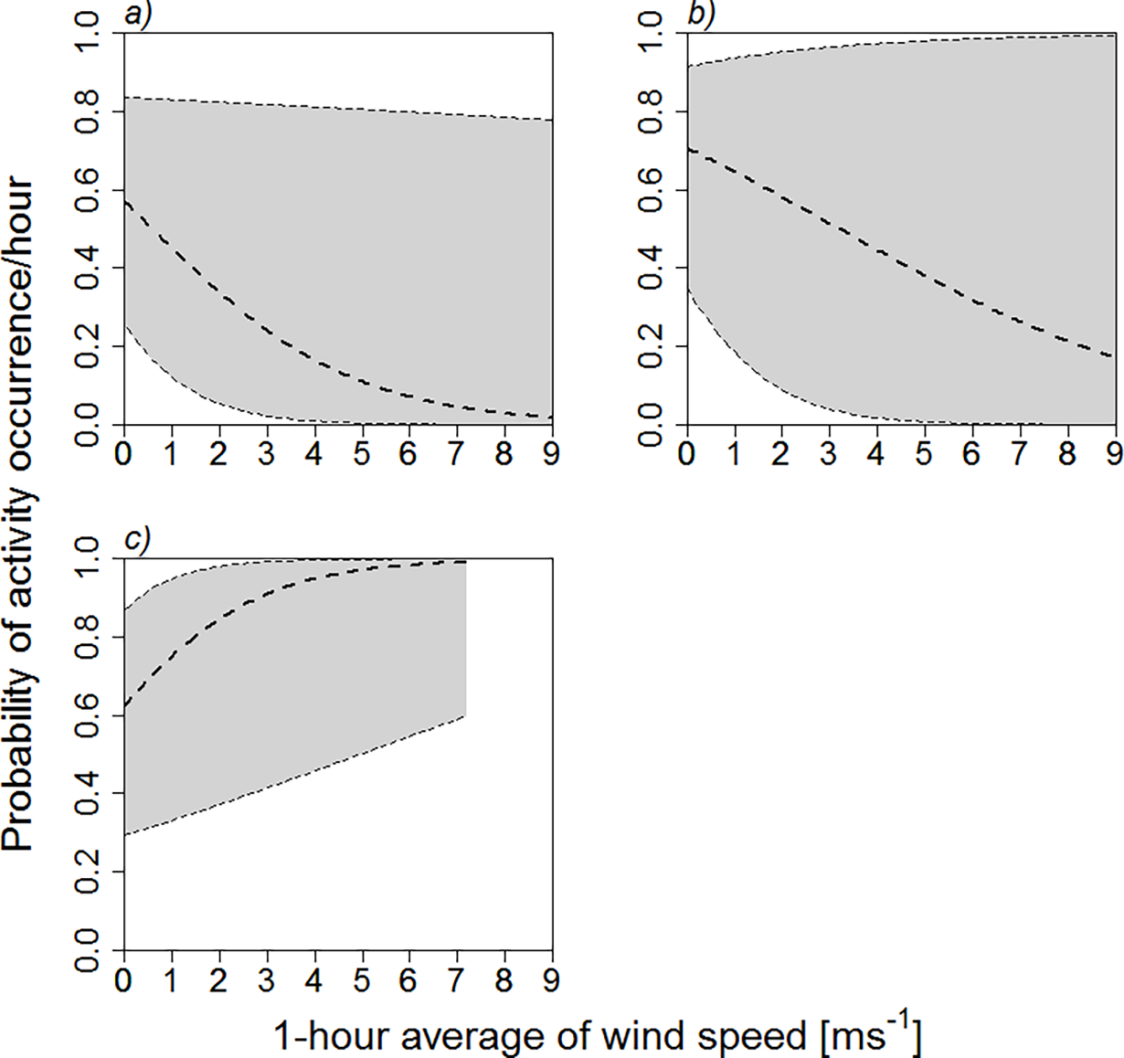
Probability of hourly bat activity occurrence in relation to mean hourly wind speed for all species pooled (mean ± 95% confidence intervals) as recorded both at the truck-mounted crane and at the six foreseen wind turbine implantation sites. Height classes. a) high: 50–65 m; b) low: 5–20 m and c) ground level (future wind turbine sites, 1 m a.g.l.).

Ground-level recordings at the six foreseen wind turbine sites indicate an unexpected increase in the probability of activity occurrence per hour with increasing wind speed up to 7 ms^-1^ (Fig 4C), which was the maximum wind speed encountered just above ground level in this study. This model was different from the null model (chi-squared χ^2^ = 8.08, df = 1, p = 0.004). The projected activity occurrence at the six sites indicates also an increase in bat activity up to 5 ms^-1^ followed by a slight decrease above 5 ms^-1^ wind speed at ground level (S8 Fig). This is further supported by the cumulated curve of bat passes per hour, which yields a 95% asymptote at 6.0 ms^-1^ (S9 Fig).

The synopsis of the ground-level data and the data obtained at the crane, remodeled for different height classes (low: 5 and 20 m and high: 50 and 65 m) suggests that there was a general shift in bat activity from higher to lower height with increasing wind speed (Fig 4).

## Discussion

This study provides quantitative assessments, from data collected at various heights above ground level, of the vertical activity profiles of a bat community occurring in an area where tall wind turbines are being deployed. It establishes which species, to which extent, are at risk of collision with rotor blades, showing in particular that one rare bat species of Southern Europe, *T*. *teniotis*, would potentially be most affected. The analysis of the relationships between bat activity and wind speed furthermore allows formulating quantitative recommendations for operating tall wind turbines in a way that would dramatically reduce collision risk for bats.

The techniques used so far for investigating bat vertical activity include bat detectors attached onto helium-filled balloons or blimps [33,34], tethered to kites [33,35], placed on meteorological, telecommunication or flux towers [36,37,38], installed in the tree canopy with a pulley system [39] or put on the jib of a crane [40]. The method used here provides bat vertical activity profiles at a higher resolution as it measures activity at five different, regularly distributed height levels (15 m increments), from ground level up to 65 m a.g.l.. The proportion of activity of the two dominant species in our bat recordings was mostly in the lower, non-dangerous zone and thus these species seem to be at relatively low risk. Although moderate to high mortality risks at wind turbines have been reported for the common pipistrelle (*P*. *pipistrellus*) and Savi’s pipistrelle (*H*. *savii*) at wind turbines across Europe [12,22,41,42,43]. A similar, even more pronounced vertical activity pattern was found for the locally rare sibling mouse-eared bats (*M*.*myotis*/*M*.*blythii)*, which appeared to be restricted to very low height, mostly just above ground level, in accordance with their mostly gleaning foraging strategy [27]. Scarce findings of dead greater mouse-eared bats (*M*. *myotis*) near wind turbines in Europe support their rather low risk of being killed at wind turbines [12]. However, both mouse-eared bats were generally rarely recorded in the study area despite there exists a mixed nursery roost 2 km from the study site. Two reasons may explain the scarce acoustic contacts we obtained. First, both species exploit farmland but have foraging territories located up to 25 km from the nursery roost, which gives very low foraging densities [29], i.e. a low probability of encounter at any given site. Second, their low-intensity echolocation calls [30,44] may also explain the scarce recordings. The species that appears to be potentially most threatened, based on both its rare regional conservation status and foraging altitude, is the European free-tailed bat (*T*. *teniotis*), whose activity was principally recorded within the dangerous rotor-swept zone within our measurements range (50–65 m a.g.l.). Moreover, the shape of the species’ vertical activity profile projection (Fig 1) suggests that a considerable, additional proportion of activity can be expected in the non-surveyed rotor-swept zone of tall turbines (65–150 m a.g.l.). This would be in line with other findings [42] that *T*. *teniotis* is a frequent victim of wind turbines in the Mediterranean.

The analysis of the relationships between bat activity and wind speed at different heights delivered detailed information for a better assessment of the risks bats incur from the development of wind energy production. The three different activity metrics used, all set in relation to mean hourly wind speed, yielded convergent results. The truck-mounted crane data showed a dramatic decrease of overall bat activity with increasing wind speed, notably above 5.4 ms^-1^ within the rotor-swept zone (Fig 2), which is in accordance with the few other European studies [12,41].

The unexpected observation that bat activity at ground level (measures obtained at the six turbine sites) increased with wind speed up to ca 5 ms^-1^, and only slightly decreased above it up to 7 ms^-1^ (the latter being the maximum wind speed measured at ground level) most probably results from a shift of bat activity towards lower heights under the pressure of accelerating wind. This is substantiated by the data collected at the crane that show a redistribution of proportional bat activity towards lower heights with increasing wind speed (Fig 4). Comparable findings were obtained in a Central European bat community [45]. In North America, activity peaked at higher wind speed [46], which might be due to numerous fast flying migrating bat species–typical in their study area–having been constrained to fly in more adverse conditions than our resident population. That aerial-hawking bats make use of the protection of vegetation shelters, for instance hedges, to forage under windy circumstances is a common fact [47]. Not only do wind-breaks facilitate bat flight, but they also enable insects to remain active in an overall windy environment.

Pre-construction estimation of the potential collision risk for bats would be crucial for the establishment of environmental impact assessments, although it is still unclear whether pre-construction bat acoustic data are able to properly predict post-construction bat fatalities. The issue remains that findings from local studies cannot be ubiquitously transferred because bat community composition usually differs between areas [48], which calls for adequate surveys of local bat communities. In our study area, for instance, the community is dominated by non-migratory bat species and there were very few recordings of migratory species, even in the late summer and fall when bat migration can be quite intense over Switzerland [23]. In fact, in the Alps, most of the autumn migration takes place at high elevation, with bats commuting mostly over mountain passes [23], while our projected wind park is situated at valley bottom level. Our observations and subsequent management recommendations would thus primarily apply to Southern European plains and valley floors with similar bat communities but not to Alpine passes and ridges where bat migration concentrates.

### Recommendations for bat-friendly wind turbine operation

Based on the status of local bat species, their vertical activity profiles and wind specific activity, we can formulate simple recommendations for operating tall wind turbines in a more bat-friendly way, as proposed by other authors [49]. In North America, increasing cut-in speed (wind speed at which wind turbines begin to produce electrical power) has been established to reduce the probability of bat fatalities [50,51]. As rotors of wind turbines typically rotate idly below cut-in speed thresholds [51], our recommendations rather refer to the start-up speed (wind speed at which the rotors begin to spin). From our results, it appears that *P*. *pipistrellus*, *H*. *savii* and *T*. *teniotis* sustain some flight activity above a wind speed of 2.5–3 ms^-1^, which is the typical cut-in-speed for operating tall wind turbines in Central Europe. Activity of *P*. *pipistrellus* and *H*. *savii* mostly occurs in the lower, non-dangerous zone, whereas the rare *T*. *teniotis* faces relatively much higher collision risks given its observed and projected activity within the dangerous zone between 50–150 m a.g.l.. Thus, the main potential impact of tall wind turbines here clearly concerns *T*. *teniotis*. As this species seems to entirely cease its activity at wind speeds > 5 ms^-1^, we propose to set a start-up speed of 5 ms^-1^ for operating tall wind turbines installed at low altitude in Southern Europe where *T*. *teniotis* occurs. This threshold also corresponds to a drop below 5% of overall bat activity within the lower part of the rotor-swept zone (<65 m a.g.l.), which would dramatically diminish collision risks for the entire bat community. Yet, we must stress that such a nighttime restriction should be implemented the year-round because *T*. *teniotis* also forages in winter, due to its very peculiar hibernation physiology [24], preying then exclusively on winter active moths.

Until efficient deterrents for bats at wind turbines are developed [52,53], it seems that regulating the start-up speed is one of the most effective measures for mitigating bat fatalities at tall wind turbines ([50,51], this study). Given the low efficiency of tall wind turbines in terms of electricity production at low wind speed and given the fact that their electricity production does not augment above approximately 11 ms^-1^ wind speed due to mechanic constraints, setting the start-up speed at 5 ms^-1^ would entail only a small loss of electricity production. In lowland areas of southern Europe where similar bat communities occur, the risks of collision with turning rotor blades likely to be experienced by bats would be drastically reduced if the electricity industry would consent a minute sacrifice in terms of productivity.

## Supporting information

S1 File Additional information about the locally three rare bat species. (PDF)

S2 File How BatScope operates. (PDF)

S1 Table Chronological order of recordings and total number of recordings per night. (PDF)

S2 Table Overview of hourly wind speed at the crane. (PDF)

S3 Table Generalized linear mixed models describing the relationship between bat activity and wind speed (metrics 1 and 2). (PDF)

S4 Table Overview of hourly number of bat passes and wind speed at the crane. (PDF)

S5 Table Overview of hourly number of bat passes and wind speed at the six projected wind turbine sites. (PDF)

S1 Fig Distribution of recording sites within the study area. Round symbols indicate recordings at foreseen wind turbine sites (ValEole 1–6; yellow: fruit tree plantations, blue: open fields). The green triangle indicates the first site for vertical recordings (Solverse). The second site (Marais d’Ardon) is situated 10 km northeast and therefore not depicted here.
(TIFF)

S2 Fig Installation system to measure vertical bat activity profiles. Left: Installation of the bat recorders on the truck-mounted crane (vertical activity profiles): two 2 cm thick metal cables were stretched from the ground level (fixed on two cement blocks of 2.5 tons each) to a 4 m long horizontal bar fixed under the crane hook, which was pulled up to 70 m above ground. Thereby the cables were kept under permanent high tension in order to avoid to whole system to twist. The distance of 4 m between the two vertical cables was maintained by horizontal metal bars (4.5 m each, positioned at 5 m, 20 m, 35 m, 50 m and 65 m a.g.l., respectively) at both ends of which the bat detectors (red squares) were fixed. Two additional recorders were attached to the uppermost bar at 70 m a.g.l., resulting in 12 recorders in total. Right: bat detector (inside the black protection box) attached at one end of a horizontal metal bar. The microphone is inside a plastic protection tube (white). During measurements microphones were directed downwards with an angle of approximately 30° to prevent damage from rain.
(TIFF)

S3 Fig Installation system with plastic sticks for ground level automatic recordings at the foreseen wind turbine sites. The microphone is inside a plastic protection tube (white) and the bat detector is inside the protection box (black). Microphones were directed downwards with an angle of approximately 30° to prevent damage from rain.
(TIFF)

S4 Fig Relative overlap (see material and methods for details) between recordings (number of bat passes) obtained at the truck-mounted crane. Left bar: recordings from the same height (SE vs NW). Right bar: recordings from two adjacent heights. Bars indicate the standard error of the mean (SE).
(EPS)

S5 Fig Evaluation of of Eqs (1) and (2) used for inter-/extrapolating wind speed measurement at different heights. Wind speed data derived from Eq (1) used at the crane (dashed line) and wind speed data derived from Eq (2) used at the six foreseen wind turbine sites (dotted line) are highly correlated with the local wind speed-height profile (solid line) in the study area.
(EPS)

S6 Fig Phenology of nocturnal bat activity (mean number of bat passes per hour, from 20 h to 5 h). Data are shown for all species pooled, as recorded at a) the truck-mounted crane and b) at ground level at the six foreseen wind turbine sites. Error bars indicate the standard error of the mean (SE).
(TIFF)

S7 Fig Projected activity occurrence per hour at the truck-mounted crane in relation to mean hourly wind speed. Models were calculated for a) all bat species pooled, b) *P*. *pipistrellus*, c) *H*. *savii*, d) *M*. *myotis/M*. *blythii* and e) *T*. *teniotis*. Black bars: all heights pooled; white bars: only heights ≥ 50 m a.g.l..
(TIFF)

S8 Fig Projected activity occurrence per hour in relation to mean hourly wind speed for all species pooled at the six foreseen wind turbine sites. (EPS)

S9 Fig Cumulative number of bat passes per hour in relation to mean hourly wind speed for all species pooled at the six foreseen wind turbine sites. The black line indicates wind speed related to 95% of the asymptote.
(EPS)
