# Supplementary material for: Mitigating the negative impacts of tall wind turbines on bats: Vertical activity profiles and relationships to wind speed
Source: PLoS One. 2018 Mar 21;13(3):e0192493. doi: 10.1371/journal.pone.0192493 (PMC5862399; doi:10.1371/journal.pone.0192493)
Supplement: S1 File — (PDF) [file pone.0192493.s001.pdf]

Supporting information

S1 File. Additional information about the locally three rare bat species.

The two sibling species *Myotis myotis* and *Myotis blythii* hunt primarily by gleaning their prey from substrates, notably bare ground, leaf litter and grass [1,2,3]. The typical foraging behaviour consists of a prospecting flight at approximately 30–70 cm above ground level; when a prey is detected, the bat lands on it, picks it up and eats it on the wing, unless the prey is too large, which necessitates discarding it from a perch [1]. *M. myotis* feeds predominantly on ground-dwelling insects such as carabid beetles (Carabidae) [1,2,3]. It forages mainly in habitats offering access to ground such as freshly mown meadows, the floor of fruit tree plantations and forests without understorey, field or grass layer [3]. *M. blythii* preys mostly on grass-dwelling arthropods, mainly bush crickets (Tettigoniidae), gleaning them from grass stalks, preferring steppe, pastureland and unmown meadows as foraging grounds [2,3]. Both uncluttered substrates granting access to the ground (fruit tree plantations with patches of bare soil and short ground vegetation) and cluttered substrates with dense vegetation cover (grasslands) occur at the projected wind park site. Next to gleaning, mouse-eared bats also hunt by aerial hawking, i.e. catching flying prey on the wing [1]. They use this hunting technique especially for cockchafers *(Melolontha melolontha)* in April-June [1,4]. While foraging, mouse-eared bats fly at moderate speed; during commuting flights, however, they can reach 50 km h^{-1} [5]. For detection and localisation of prey, *M. myotis* and *M. blythii* rely on passive listening when gleaning and ultrasonic echolocation during aerial hawking [6,7]. While bats are passively listening for prey-generated sounds, they do constantly echolocate for orientation in space but calls are weak with low amplitudes ("whispering echolocation"; see [6,7]). For *M. myotis* the call frequency of highest energy (FMAXE) lies at 31–54 kHz, for *M. blythii* at 33–52 kHz [8].

Due to its high body mass and narrow wings (high aspect ratio), *T. teniotis* presents morphological characteristics typical of a fast hawking bat species, reaching up to 50 km h^{-1} average speed on commuting flights, with probably peaks up to 80 km h^{-1} (Arlettaz, unpublished). It forages in the open air space, hawking on large tympanate insects of the orders Lepidoptera and Neuroptera [9]. *T. teniotis* undertake long-lasting foraging bouts during the night, covering long commuting distances between roosts and foraging grounds (up to 80 km in the same night; Arlettaz, unpublished). For detecting and locating its prey *T. teniotis* uses low frequency echolocation calls (11–18 kHz), which are audible for humans. Low frequency calls enable *T. teniotis* to feed on tympanate insects (the allotonic frequency hypothesis), whose hearing apparatus cannot detect such low frequencies [9]. *T. teniotis* undertakes partial hibernation in winter, engaging in short phases of lethargy, but its peculiar physiology makes that it cannot sustain long periods at roost temperatures below 7.5°C; below this threshold its energy expenditure dramatically increases [10]. Unlike most other bat species in the study area, *T. teniotis* has to forage during winter to regularly refuel its fat reserves [10]. It then relies on several species of moths that have an exclusive winter phenology.

References

1. Arlettaz R. Feeding behaviour and foraging strategy of free-living mouse-eared bats, *Myotis myotis* and *Myotis blythii*. Animal Behaviour. 1996;51:1-11.
2. Arlettaz R, Perrin N, Hausser J. Trophic resource partitioning and competition between the two sibling bat species *Myotis myotis* and *Myotis blythii*. Journal of Animal Ecology. 1997;66(6):897-911.
3. Arlettaz R. Habitat selection as a major resource partitioning mechanism between the two sympatric sibling bat species *Myotis myotis* and *Myotis blythii*. Journal of Animal Ecology. 1999;68(3):460-71.
4. Arlettaz R, Christe P, Lugon A, Perrin N, Vogel P. Food availability dictates the timing of parturition in insectivorous mouse-eared bats. Oikos. 2001;95(1):105-11.
5. Arlettaz R. Ecology of the sibling mouse-eared bats (*Myotis myotis* and *Myotis blythii*): zoogeography, niche, competition, and foraging [Ph.D. thesis]: University of Lausanne, Switzerland; 1995.
6. Arlettaz R, Jones G, Racey PA. Effect of acoustic clutter on prey detection by bats. Nature. 2001;414(6865):742-5.
7. Russo D, Jones G, Arlettaz R. Echolocation and passive listening by foraging mouse-eared bats *Myotis myotis* and *M. blythii*. Journal of Experimental Biology. 2007;210(1):166-76.
8. Russo D, Jones G. Identification of twenty-two bat species *(Mammalia* : *Chiroptera*) from Italy by analysis of time-expanded recordings of echolocation calls. Journal of Zoology. 2002;258:91-103.
9. Rydell J, Arlettaz R. Low-frequency echolocation enables the bat *Tadarida teniotis* to feed on tympanate insects. Proceedings of the Royal Society B-Biological Sciences. 1994;257(1349):175-8.
10. Arlettaz R, Ruchet C, Aeschimann J, Brun E, Genoud M, Vogel P. Physiological traits affecting the distribution and wintering strategy of the bat *Tadarida teniotis*. Ecology. 2000;81(4):1004-14.
