# Supplementary material for: Mitigating the negative impacts of tall wind turbines on bats: Vertical activity profiles and relationships to wind speed
Source: PLoS One. 2018 Mar 21;13(3):e0192493. doi: 10.1371/journal.pone.0192493 (PMC5862399; doi:10.1371/journal.pone.0192493)
Supplement: S2 File — (PDF) [file pone.0192493.s002.pdf]

Supporting information

S2 File. How BatScope operates

In a first step, the software cuts out any detected bat calls from the sequences. In a second step, it classifies single calls comparing 23 measured parameters with a reference library of 20'00 calls. Built-in classifiers employed are a Support Vector Machine (SVM), a K Nearest Neighbour (KNN) classifier, and a Quadratic Discriminant Analysis (QDA). Each call within a sequence is thus assigned to a most likely bat species, by probabilistically including the output of the three classifiers. As double control, after a species has been proposed, BatScope tests the three most distinctive features i.e. call duration, peak frequency and bandwidth of a call against reference values of the proposed species. For a classification to be accepted all values have to lie within a 95% confidence interval of the reference values. BatScope summarizes classification results of single calls and from these, calculates overall statistics for each sequence. Thus, each sequence gets assigned to a species with a given classification probability [1]. Since not all of these species are likely to be found in the lower Rhône Valley and some are almost impossible to be correctly acoustically distinguished from others, we combined some species, creating 15 groups of bat species for further analysis. Only decent quality recordings containing bat calls with a signal-to-noise ratio (SNR) of at least 30 dB were taken into account. In a final verification step we applied a filter to the classification probability (= quality), accepting classifications with probabilities ≥ 80%. Sequences with a quality < 80% were manually controlled and verified visually.

References

1. Boesch R, Obrist MK. BatScope - Implementation of a BioAcoustic Taxon Identification Tool. Swiss Federal Research Institute WSL, Birmensdorf, Switzerland; 2013.
