# Supplementary material for: Mitigating the negative impacts of tall wind turbines on bats: Vertical activity profiles and relationships to wind speed
Source: PLoS One. 2018 Mar 21;13(3):e0192493. doi: 10.1371/journal.pone.0192493 (PMC5862399; doi:10.1371/journal.pone.0192493)
Supplement: S1 Table — (PDF) [file pone.0192493.s003.pdf]

Supporting information

S1 Table. Chronological order of recordings and total number of recordings per night. Ground-level recordings at the six projected wind turbine sites (ValEole 1-6) within plantations (P) and in open fields (O), and vertical recordings (averaged per height) at Solverse (SOL) and Marais d'Ardon (MAR).

Date	ValEole1		ValEole2		ValEole3		ValEole4		ValEole5		ValEole6		SOLVERSE	MARAIS
	E: 7° 8.841' N: 46° 7.933'		E: 7° 8.442' N: 46° 7.857		E: 7° 8.538' N: 46° 8.243'		E: 7° 8.421' N: 46° 8.494'		E: 7° 8.905' N: 46° 8.488'		E: 7° 9.591' N: 46° 8.855'		E: 7° 8.841' N: 46° 7.933'	E: 7° 15.469' N: 46° 11.790'
	P	O	P	O	P	O	P	O	P	O	P	O		
12_Jul_11	34	19	21	22	12	14	15	21	14	20	11	7		
15_Jul_11													89	
16_Jul_11													130.5	
18_Jul_11	2	7	5	6	15	10	10	102	0	20	4	2		
21_Jul_11	0	6	4	10	7	4	0	158	0	18	1	1		
01_Aug_11	0	7	15	33	1	21	0	33	0	6	6	4		
04_Aug_11													14	
07_Aug_11													24.5	
10_Aug_11	11	44	7	34	7	6	0	231	0	7	0	3		
11_Aug_11	6	18	11	49	16	8	0	88	1	1	1	6		
19_Aug_11														202
20_Aug_11														148.5
21_Aug_11														230.5
28_Aug_11	17	21	20	104	3	9	11	88	37	9	0	3		
01_Sep_11	1	5	18	85	10	40	0	104	52	4	1	9		
06_Sep_11	0	6	37	160	5	13	0	50	16	4	0	59		
30_Sep_11														16
02_Oct_11														22
14_Oct_11	0	3	1	0	0	0	0	0	0	0	0	0		
15_Oct_11	0	0	0	0	0	0	0	0	0	0	0	0		
03_May_12	1	1	0	12	1	0	0	0	0	0	0	0		
07_May_12	0	0	0	3	1	0	0	28	0	0	0	1		
16_May_12	0	0	0	0	0	0	0	0	0	0	0	0		
24_May_12	2	2	0	6	0	0	0	0	0	0	0	0		
13_Jun_12	0	0	1	1	0	0	0	0	0	0	0	0		
17_Jun_12	0	0	0	2	0	6	0	47	0	0	0	5		
26_Jun_12	0	0	0	1	0	0	0	144	0	0	0	1		
29_Jun_12	0	0	0	2	1	0	0	22	0	0	0	1		
