# Supplementary material for: Mitigating the negative impacts of tall wind turbines on bats: Vertical activity profiles and relationships to wind speed
Source: PLoS One. 2018 Mar 21;13(3):e0192493. doi: 10.1371/journal.pone.0192493 (PMC5862399; doi:10.1371/journal.pone.0192493)
Supplement: S2 Table — (PDF) [file pone.0192493.s004.pdf]

Supporting information

S2 Table. Overview of hourly wind speed at the crane. Wind speed was measured at heights 10 m a.g.l. (h10) and 70 m a.g.l. (h70).

Date	Hour	Wind speed h10 [m/s]	Wind speed h70 [m/s]
15.07.2011	01:00:00	1.900	2.513
15.07.2011	02:00:00	0.883	1.800
15.07.2011	03:00:00	0.800	1.050
15.07.2011	04:00:00	0.633	1.250
15.07.2011	05:00:00	0.750	0.917
15.07.2011	06:00:00	0.650	1.533
16.07.2011	21:00:00	3.900	7.500
16.07.2011	22:00:00	3.167	5.283
16.07.2011	23:00:00	2.617	4.800
16.07.2011	00:00:00	4.917	5.017
16.07.2011	01:00:00	4.050	7.033
16.07.2011	02:00:00	2.950	5.550
16.07.2011	03:00:00	3.217	6.550
16.07.2011	04:00:00	2.350	3.900
16.07.2011	05:00:00	1.700	4.150
16.07.2011	06:00:00	1.633	4.617
16.07.2011	07:00:00	1.783	3.617
04.08.2011	20:00:00	6.460	9.067
04.08.2011	21:00:00	6.267	8.967
04.08.2011	22:00:00	6.233	9.133
04.08.2011	23:00:00	5.483	8.383
04.08.2011	00:00:00	5.467	8.117
04.08.2011	01:00:00	5.850	9.183
04.08.2011	02:00:00	3.917	7.033
04.08.2011	03:00:00	3.983	7.150
04.08.2011	04:00:00	1.067	1.867
04.08.2011	05:00:00	1.083	1.200
04.08.2011	06:00:00	1.100	1.500
07.08.2011	21:00:00	1.867	3.450
07.08.2011	22:00:00	1.467	3.733
07.08.2011	23:00:00	1.017	1.633
07.08.2011	00:00:00	0.850	1.133
07.08.2011	01:00:00	1.533	1.900
07.08.2011	02:00:00	0.750	1.800
07.08.2011	03:00:00	0.783	1.167
07.08.2011	04:00:00	0.933	0.650
07.08.2011	05:00:00	1.233	1.367
19.08.2011	21:00:00	1.917	3.417
19.08.2011	22:00:00	1.067	2.133
19.08.2011	23:00:00	0.817	1.033
19.08.2011	00:00:00	0.750	1.417
19.08.2011	01:00:00	1.050	2.383
19.08.2011	02:00:00	1.467	3.400
19.08.2011	03:00:00	1.583	3.300
19.08.2011	04:00:00	0.717	2.200
19.08.2011	05:00:00	0.817	2.083
19.08.2011	06:00:00	0.883	2.633

S5 Table continued

20.08.2011	22:00:00	1.383	2.450
20.08.2011	23:00:00	1.017	0.950
20.08.2011	00:00:00	0.800	2.100
20.08.2011	01:00:00	1.350	3.383
20.08.2011	02:00:00	0.917	3.167
20.08.2011	03:00:00	0.983	3.317
20.08.2011	04:00:00	0.767	3.617
20.08.2011	05:00:00	0.900	4.133
20.08.2011	06:00:00	0.867	3.817
21.08.2011	22:00:00	1.267	2.167
21.08.2011	23:00:00	1.683	2.933
21.08.2011	00:00:00	1.433	3.433
21.08.2011	01:00:00	1.100	2.983
21.08.2011	02:00:00	0.633	1.933
21.08.2011	03:00:00	0.733	1.667
21.08.2011	04:00:00	0.833	2.350
21.08.2011	05:00:00	1.000	3.400
21.08.2011	06:00:00	0.533	3.350
30.09.2011	21:00:00	1.033	1.167
30.09.2011	22:00:00	0.517	0.883
30.09.2011	23:00:00	0.467	0.667
30.09.2011	00:00:00	0.683	1.733
30.09.2011	01:00:00	0.717	2.950
30.09.2011	02:00:00	0.650	2.067
30.09.2011	03:00:00	0.517	1.267
30.09.2011	04:00:00	0.717	1.850
30.09.2011	05:00:00	0.850	2.717
30.09.2011	06:00:00	0.883	3.467
30.09.2011	07:00:00	1.033	3.567
02.10.2011	20:00:00	1.833	5.067
02.10.2011	21:00:00	1.717	4.267
02.10.2011	22:00:00	1.050	0.933
02.10.2011	23:00:00	0.933	1.950
02.10.2011	00:00:00	0.350	0.917
02.10.2011	01:00:00	0.567	2.133
02.10.2011	02:00:00	0.683	2.950
02.10.2011	03:00:00	0.967	4.133
02.10.2011	04:00:00	0.767	3.083
02.10.2011	05:00:00	0.350	2.283
02.10.2011	06:00:00	0.383	2.050
02.10.2011	07:00:00	0.483	2.800
