# Supplementary material for: Mitigating the negative impacts of tall wind turbines on bats: Vertical activity profiles and relationships to wind speed
Source: PLoS One. 2018 Mar 21;13(3):e0192493. doi: 10.1371/journal.pone.0192493 (PMC5862399; doi:10.1371/journal.pone.0192493)
Supplement: S3 Table — (PDF) [file pone.0192493.s005.pdf]

Supporting information

S3 Table. Generalized linear mixed models describing the relationship between bat activity and wind speed (metrics 1 and 2). GLMMs included bat activity (presence/absence) as dependent variable with wind speed as explanatory variable and recording night as a random factor. Modelling was carried out for all recorded bat species, the two most common recorded species *P. pipistrellus* and *H. savii* and the rare species *M. myotis*/*M. blythii* and *T. teniotis* on the crane for all heights (5-65 m) and for the dangerous zone only (50-65 m). The same modelling approach was applied to activity data of all bat species obtained from ground level at the six foreseen wind turbine sites.

Bat species	Data origin	Height	Fixed effects	Estimate	Std. Error	z value	p value
All bat species	Crane	All heights	Intercept	0.785	0.639	1.109	0.267
			Wind speed	-0.828	0.159	-5.214	**<0.001**
		Dangerous zone	Intercept	-0.034	0.721	-0.048	0.962
			Wind speed	-0.530	0.227	-2.337	**0.019**
	Ground		Intercept	0.504	0.841	0.600	0.549
			Wind speed	0.603	0.259	2.331	**0.020**
P. pipistrellus	Crane	All heights	Intercept	0.594	0.684	0.868	0.386
			Wind speed	-1.124	0.191	-5.802	**<0.001**
		Dangerous zone	Intercept	-0.578	1.251	-0.462	0.644
			Wind speed	-1.133	0.486	-2.334	**0.020**
H. savii	Crane	All heights	Intercept	-1.952	0.002	-1008.2	**<0.001**
			Wind speed	-0.577	0.002	-297.7	**<0.001**
		Dangerous zone	Intercept	-2.031	0.805	-2.524	**0.012**
			Wind speed	-0.432	0.396	-1.091	0.275
M. myotis/M. blythii	Crane	All heights	Intercept	-3.310	0.718	-4.608	**<0.001**
			Wind speed	-0.258	0.245	-1.053	0.292
		Dangerous zone	Intercept	-12.108	7.934	-1.526	0.127
			Wind speed	1.175	0.958	1.227	0.220
T. teniotis	Crane	All heights	Intercept	-1.891	0.002	-936.2	**<0.001**
			Wind speed	-0.572	0.002	-283.3	**<0.001**
		Dangerous zone	Intercept	-1.451	0.801	-1.811	0.070
			Wind speed	-0.458	0.313	-1.467	0.142
