# Supplementary material for: Mitigating the negative impacts of tall wind turbines on bats: Vertical activity profiles and relationships to wind speed
Source: PLoS One. 2018 Mar 21;13(3):e0192493. doi: 10.1371/journal.pone.0192493 (PMC5862399; doi:10.1371/journal.pone.0192493)
Supplement: S4 Table — (PDF) [file pone.0192493.s006.pdf]

Supporting information

S4 Table. Overview of hourly number of bat passes and wind speed at the crane.

Date	Hour	Height [m]	Total bat passes	Averaged bat passes	Averaged bat passes *P. pipistrellus*	Averaged bat passes *H. savii*	Averaged bat passes *M. myotis/M. blythii*	Averaged bat passes *T. teniotis*	Wind speed [m/s]
15.07.2011	01:00:00	5	12	6	6	0	0	0	1.682
15.07.2011	02:00:00	5	18	9	7.5	0	0	0	0.556
15.07.2011	03:00:00	5	3	1.5	1	0	0	0	0.711
15.07.2011	04:00:00	5	0	0	0	0	0	0	0.413
15.07.2011	05:00:00	5	2	1	0.5	0	0	0.5	0.691
16.07.2011	22:00:00	5	12	6	2.5	2.5	0	0	2.413
16.07.2011	23:00:00	5	61	30.5	15	14	1.5	0	1.839
16.07.2011	00:00:00	5	11	5.5	4	1.5	0	0	4.881
16.07.2011	01:00:00	5	6	3	2	1	0	0	2.987
16.07.2011	02:00:00	5	23	11.5	10	1.5	0	0	2.024
16.07.2011	03:00:00	5	17	8.5	6.5	2	0	0	2.030
16.07.2011	04:00:00	5	14	7	4.5	3	0	0	1.798
16.07.2011	05:00:00	5	8	4	3	0.5	0	0.5	0.827
04.08.2011	22:00:00	5	0	0	0	0	0	0	5.200
04.08.2011	23:00:00	5	1	0.5	0	0.5	0	0	4.450
04.08.2011	00:00:00	5	1	0.5	0.5	0	0	0	4.523
04.08.2011	01:00:00	5	0	0	0	0	0	0	4.663
04.08.2011	02:00:00	5	1	0.5	0.5	0	0	0	2.807
04.08.2011	03:00:00	5	0	0	0	0	0	0	2.855
04.08.2011	04:00:00	5	5	2.5	1.5	0.5	0	0	0.782
04.08.2011	05:00:00	5	1	0.5	0.5	0	0	0	1.041
07.08.2011	22:00:00	5	14	7	3	3	0	0	0.660
07.08.2011	23:00:00	5	1	0.5	0.5	0	0	0	0.798
07.08.2011	00:00:00	5	1	0.5	0.5	0	0	0	0.749
07.08.2011	01:00:00	5	1	0.5	0.5	0	0	0	1.402
07.08.2011	02:00:00	5	1	0.5	0.5	0	0	0	0.376
07.08.2011	03:00:00	5	0	0	0	0	0	0	0.646
07.08.2011	04:00:00	5	0	0	0	0	0	0	1.034
07.08.2011	05:00:00	5	0	0	0	0	0	0	1.185
19.08.2011	22:00:00	5	121	60.5	43	12.5	2	0	0.687
19.08.2011	23:00:00	5	21	10.5	5.5	4	1	0	0.740
19.08.2011	00:00:00	5	12	6	3.5	1.5	1	0.5	0.512
19.08.2011	01:00:00	5	9	4.5	3	0	0	0	0.575
19.08.2011	02:00:00	5	13	6.5	3.5	0	0	0	0.778
19.08.2011	03:00:00	5	12	6	5	0	1	0	0.971
19.08.2011	04:00:00	5	10	5	4.5	0	0	0	0.189
19.08.2011	05:00:00	5	2	1	0.5	0	0	0	0.366
20.08.2011	22:00:00	5	80	40	30	6.5	1	0	1.003
20.08.2011	23:00:00	5	31	15.5	9.5	3	0.5	0	1.041
20.08.2011	00:00:00	5	9	4.5	3.5	0.5	0	0	0.337
20.08.2011	01:00:00	5	8	4	3.5	0	0	0	0.626
20.08.2011	02:00:00	5	13	6.5	5.5	0.5	0	0	0.116
20.08.2011	03:00:00	5	10	5	4	0	0	0	0.152
20.08.2011	04:00:00	5	4	2	2	0	0	0	0
20.08.2011	05:00:00	5	0	0	0	0	0	0	0
21.08.2011	22:00:00	5	89	44.5	29.5	4	0	0	0.946
21.08.2011	23:00:00	5	17	8.5	5	0	0.5	1.5	1.238
21.08.2011	00:00:00	5	27	13.5	11	1	0	0	0.721
21.08.2011	01:00:00	5	13	6.5	6	0	0	0	0.429
21.08.2011	02:00:00	5	18	9	7	0.5	0	1	0.170
21.08.2011	03:00:00	5	27	13.5	9	0	0	3.5	0.400
21.08.2011	04:00:00	5	19	9.5	8.5	0	0.5	0	0.293
21.08.2011	05:00:00	5	16	8	5.5	0	0	2.5	0.145
30.09.2011	21:00:00	5	5	2.5	2.5	0	0	0	0.985
30.09.2011	22:00:00	5	2	1	1	0	0	0	0.387
30.09.2011	23:00:00	5	0	0	0	0	0	0	0.396
30.09.2011	00:00:00	5	0	0	0	0	0	0	0.309
30.09.2011	01:00:00	5	0	0	0	0	0	0	0
30.09.2011	02:00:00	5	0	0	0	0	0	0	0.145
30.09.2011	03:00:00	5	0	0	0	0	0	0	0.250
30.09.2011	04:00:00	5	0	0	0	0	0	0	0.313
30.09.2011	05:00:00	5	0	0	0	0	0	0	0.185
30.09.2011	06:00:00	5	0	0	0	0	0	0	0
30.09.2011	07:00:00	5	0	0	0	0	0	0	0.130
02.10.2011	21:00:00	5	2	1	0.5	0	0	0	0.809
02.10.2011	22:00:00	5	1	0.5	0.5	0	0	0	1.092
02.10.2011	23:00:00	5	0	0	0	0	0	0	0.571
02.10.2011	00:00:00	5	0	0	0	0	0	0	0.148
02.10.2011	01:00:00	5	0	0	0	0	0	0	0.009
02.10.2011	02:00:00	5	0	0	0	0	0	0	0
02.10.2011	03:00:00	5	0	0	0	0	0	0	0
02.10.2011	04:00:00	5	0	0	0	0	0	0	0
02.10.2011	05:00:00	5	0	0	0	0	0	0	0
02.10.2011	06:00:00	5	0	0	0	0	0	0	0
02.10.2011	07:00:00	5	0	0	0	0	0	0	0

S3 Table continued

15.07.2011	01:00:00	20	11	5.5	5.5	0	0	0	2.118
15.07.2011	02:00:00	20	14	7	6	0.5	0	0	1.21
15.07.2011	03:00:00	20	9	4.5	3.5	0.5	0	0	0.889
15.07.2011	04:00:00	20	1	0.5	0.5	0	0	0	0.853
15.07.2011	05:00:00	20	2	1	0.5	0	0	0.5	0.809
16.07.2011	22:00:00	20	8	4	1.5	1	0	0	3.921
16.07.2011	23:00:00	20	30	15	7.5	5	0	0	3.395
16.07.2011	00:00:00	20	3	1.5	1.5	0	0	0	4.953
16.07.2011	01:00:00	20	1	0.5	0	0.5	0	0	5.113
16.07.2011	02:00:00	20	10	5	4.5	0.5	0	0	3.876
16.07.2011	03:00:00	20	2	1	1	0	0	0	4.404
16.07.2011	04:00:00	20	8	4	1.5	3	0	0	2.902
16.07.2011	05:00:00	20	6	3	1	1	0	1	2.573
04.08.2011	22:00:00	20	2	1	0.5	0.5	0	0	7.266
04.08.2011	23:00:00	20	1	0.5	0	0.5	0	0	6.516
04.08.2011	00:00:00	20	0	0	0	0	0	0	6.411
04.08.2011	01:00:00	20	0	0	0	0	0	0	7.037
04.08.2011	02:00:00	20	0	0	0	0	0	0	5.027
04.08.2011	03:00:00	20	0	0	0	0	0	0	5.111
04.08.2011	04:00:00	20	0	0	0	0	0	0	1.352
04.08.2011	05:00:00	20	2	1	1	0	0	0	1.125
07.08.2011	22:00:00	20	0	0	0	0	0	0	2.274
07.08.2011	23:00:00	20	1	0.5	0.5	0	0	0	1.236
07.08.2011	00:00:00	20	0	0	0	0	0	0	0.951
07.08.2011	01:00:00	20	0	0	0	0	0	0	1.664
07.08.2011	02:00:00	20	0	0	0	0	0	0	1.124
07.08.2011	03:00:00	20	0	0	0	0	0	0	0.92
07.08.2011	04:00:00	20	0	0	0	0	0	0	0.832
07.08.2011	05:00:00	20	0	0	0	0	0	0	1.281
19.08.2011	22:00:00	20	39	19.5	16.5	3	0	0	1.447
19.08.2011	23:00:00	20	2	1	1	0	0	0	0.894
19.08.2011	00:00:00	20	0	0	0	0	0	0	0.988
19.08.2011	01:00:00	20	1	0.5	0.5	0	0	0	1.525
19.08.2011	02:00:00	20	4	2	0.5	0	0	0	2.156
19.08.2011	03:00:00	20	1	0.5	0.5	0	0	0	2.195
19.08.2011	04:00:00	20	0	0	0	0	0	0	1.245
19.08.2011	05:00:00	20	0	0	0	0	0	0	1.268
20.08.2011	22:00:00	20	9	4.5	3.5	0.5	0	0	1.763
20.08.2011	23:00:00	20	3	1.5	1.5	0	0	0	0.993
20.08.2011	00:00:00	20	4	2	1.5	0	0	0	1.263
20.08.2011	01:00:00	20	1	0.5	0.5	0	0	0	2.074
20.08.2011	02:00:00	20	0	0	0	0	0	0	1.718
20.08.2011	03:00:00	20	1	0.5	0.5	0	0	0	1.814
20.08.2011	04:00:00	20	0	0	0	0	0	0	1.782
20.08.2011	05:00:00	20	0	0	0	0	0	0	2.052
21.08.2011	22:00:00	20	40	20	6.5	6.5	0	0	1.588
21.08.2011	23:00:00	20	13	6.5	2	0	1.5	3	2.128
21.08.2011	00:00:00	20	14	7	4	2	0	0	2.145
21.08.2011	01:00:00	20	8	4	4	0	0	0	1.771
21.08.2011	02:00:00	20	15	7.5	5.5	0	0	2	1.096
21.08.2011	03:00:00	20	26	13	14	0.5	0	3.5	1.066
21.08.2011	04:00:00	20	14	7	6.5	0	0	0	1.373
21.08.2011	05:00:00	20	12	6	4	0	0	2	1.855
30.09.2011	21:00:00	20	5	2.5	2	0	0	0	1.081
30.09.2011	22:00:00	20	4	2	2	0	0	0	0.647
30.09.2011	23:00:00	20	0	0	0	0	0	0	0.538
30.09.2011	00:00:00	20	0	0	0	0	0	0	1.057
30.09.2011	01:00:00	20	0	0	0	0	0	0	1.512
30.09.2011	02:00:00	20	1	0.5	0.5	0	0	0	1.155
30.09.2011	03:00:00	20	0	0	0	0	0	0	0.784
30.09.2011	04:00:00	20	0	0	0	0	0	0	1.121
30.09.2011	05:00:00	20	0	0	0	0	0	0	1.515
30.09.2011	06:00:00	20	0	0	0	0	0	0	1.803
30.09.2011	07:00:00	20	0	0	0	0	0	0	1.936
02.10.2011	21:00:00	20	1	0.5	0.5	0	0	0	2.625
02.10.2011	22:00:00	20	6	3	2	0.5	0	0	1.008
02.10.2011	23:00:00	20	7	3.5	2	0	0	0	1.295
02.10.2011	00:00:00	20	11	5.5	4.5	0	0	0	0.552
02.10.2011	01:00:00	20	2	1	0	0	0	0	1.125
02.10.2011	02:00:00	20	4	2	1	0	0	0	1.491
02.10.2011	03:00:00	20	4	2	0.5	0	0	0	2.095
02.10.2011	04:00:00	20	0	0	0	0	0	0	1.592
02.10.2011	05:00:00	20	5	2.5	1	0	0	1	1.039
02.10.2011	06:00:00	20	0	0	0	0	0	0	0.977
02.10.2011	07:00:00	20	0	0	0	0	0	0	1.308

S3 Table continued

15.07.2011	01:00:00	35	3	1.5	1.5	0	0	0	2.294
15.07.2011	02:00:00	35	6	3	2	0.5	0	0	1.474
15.07.2011	03:00:00	35	4	2	0.5	0	0	0	0.961
15.07.2011	04:00:00	35	2	1	1	0	0	0	1.030
15.07.2011	05:00:00	35	4	2	1.5	0	0	0.5	0.857
16.07.2011	22:00:00	35	5	2.5	0	0.5	0	0	4.529
16.07.2011	23:00:00	35	5	2.5	1	0	0	0	4.023
16.07.2011	00:00:00	35	4	2	1	0	0	0	4.982
16.07.2011	01:00:00	35	1	0.5	0	0.5	0	0	5.971
16.07.2011	02:00:00	35	1	0.5	0	0.5	0	0	4.624
16.07.2011	03:00:00	35	0	0	0	0	0	0	5.362
16.07.2011	04:00:00	35	2	1	0	1	0	0	3.348
16.07.2011	05:00:00	35	2	1	0	0.5	0	0.5	3.277
04.08.2011	22:00:00	35	0	0	0	0	0	0	8.100
04.08.2011	23:00:00	35	0	0	0	0	0	0	7.350
04.08.2011	00:00:00	35	0	0	0	0	0	0	7.173
04.08.2011	01:00:00	35	0	0	0	0	0	0	7.995
04.08.2011	02:00:00	35	0	0	0	0	0	0	5.923
04.08.2011	03:00:00	35	0	0	0	0	0	0	6.022
04.08.2011	04:00:00	35	1	0.5	0	0.5	0	0	1.582
04.08.2011	05:00:00	35	2	1	1	0	0	0	1.159
07.08.2011	22:00:00	35	1	0.5	0	0.5	0	0	2.925
07.08.2011	23:00:00	35	0	0	0	0	0	0	1.413
07.08.2011	00:00:00	35	0	0	0	0	0	0	1.032
07.08.2011	01:00:00	35	0	0	0	0	0	0	1.770
07.08.2011	02:00:00	35	0	0	0	0	0	0	1.426
07.08.2011	03:00:00	35	0	0	0	0	0	0	1.030
07.08.2011	04:00:00	35	0	0	0	0	0	0	0.751
07.08.2011	05:00:00	35	0	0	0	0	0	0	1.320
19.08.2011	22:00:00	35	50	25	12	13.5	0	0	1.753
19.08.2011	23:00:00	35	17	8.5	3.5	4.5	0	0	0.956
19.08.2011	00:00:00	35	9	4.5	0.5	0.5	0	2	1.180
19.08.2011	01:00:00	35	2	1	0.5	0	0	0	1.908
19.08.2011	02:00:00	35	22	11	2.5	0	0	0	2.712
19.08.2011	03:00:00	35	0	0	0	0	0	0	2.689
19.08.2011	04:00:00	35	2	1	1	0	0	0	1.671
19.08.2011	05:00:00	35	0	0	0	0	0	0	1.632
20.08.2011	22:00:00	35	32	16	6.5	8	0	0	2.070
20.08.2011	23:00:00	35	10	5	1.5	2.5	0	0	0.974
20.08.2011	00:00:00	35	8	4	2.5	0.5	0	0	1.637
20.08.2011	01:00:00	35	1	0.5	0.5	0	0	0	2.658
20.08.2011	02:00:00	35	1	0.5	0	0.5	0	0	2.365
20.08.2011	03:00:00	35	2	1	0.5	0	0	0	2.485
20.08.2011	04:00:00	35	1	0.5	0.5	0	0	0	2.601
20.08.2011	05:00:00	35	1	0.5	0	0	0	0	2.981
21.08.2011	22:00:00	35	19	9.5	3	3	0	2	1.847
21.08.2011	23:00:00	35	11	5.5	1.5	0	0.5	1	2.487
21.08.2011	00:00:00	35	8	4	2.5	1.5	0	0	2.720
21.08.2011	01:00:00	35	1	0.5	0	0	0	0	2.312
21.08.2011	02:00:00	35	7	3.5	1	0	0	2.5	1.470
21.08.2011	03:00:00	35	4	2	0	0	0	2	1.335
21.08.2011	04:00:00	35	2	1	0	0	0	1	1.809
21.08.2011	05:00:00	35	1	0.5	0.5	0	0	0	2.545
30.09.2011	21:00:00	35	1	0.5	0.5	0	0	0	1.120
30.09.2011	22:00:00	35	0	0	0	0	0	0	0.752
30.09.2011	23:00:00	35	0	0	0	0	0	0	0.596
30.09.2011	00:00:00	35	0	0	0	0	0	0	1.359
30.09.2011	01:00:00	35	0	0	0	0	0	0	2.154
30.09.2011	02:00:00	35	0	0	0	0	0	0	1.562
30.09.2011	03:00:00	35	0	0	0	0	0	0	1.000
30.09.2011	04:00:00	35	0	0	0	0	0	0	1.447
30.09.2011	05:00:00	35	0	0	0	0	0	0	2.052
30.09.2011	06:00:00	35	0	0	0	0	0	0	2.546
30.09.2011	07:00:00	35	0	0	0	0	0	0	2.665
02.10.2011	21:00:00	35	1	0.5	0.5	0	0	0	3.358
02.10.2011	22:00:00	35	0	0	0	0	0	0	0.974
02.10.2011	23:00:00	35	0	0	0	0	0	0	1.587
02.10.2011	00:00:00	35	0	0	0	0	0	0	0.715
02.10.2011	01:00:00	35	0	0	0	0	0	0	1.575
02.10.2011	02:00:00	35	0	0	0	0	0	0	2.143
02.10.2011	03:00:00	35	0	0	0	0	0	0	3.005
02.10.2011	04:00:00	35	0	0	0	0	0	0	2.258
02.10.2011	05:00:00	35	0	0	0	0	0	0	1.595
02.10.2011	06:00:00	35	0	0	0	0	0	0	1.456
02.10.2011	07:00:00	35	0	0	0	0	0	0	1.974

S3 Table continued

15.07.2011	01:00:00	50	5	2.5	2.5	0	0	0	2.407
15.07.2011	02:00:00	50	7	3.5	2.5	1	0	0	1.642
15.07.2011	03:00:00	50	11	5.5	5.5	0	0	0	1.007
15.07.2011	04:00:00	50	7	3.5	3.5	0	0	0	1.143
15.07.2011	05:00:00	50	12	6	4	0	0	2	0.888
16.07.2011	22:00:00	50	1	0.5	0	0.5	0	0	4.917
16.07.2011	23:00:00	50	5	2.5	0.5	1	0	0	4.423
16.07.2011	00:00:00	50	2	1	0.5	0	0	0	5
16.07.2011	01:00:00	50	0	0	0	0	0	0	6.518
16.07.2011	02:00:00	50	0	0	0	0	0	0	5.101
16.07.2011	03:00:00	50	0	0	0	0	0	0	5.973
16.07.2011	04:00:00	50	0	0	0	0	0	0	3.632
16.07.2011	05:00:00	50	4	2	0	0.5	0	1.5	3.726
04.08.2011	22:00:00	50	0	0	0	0	0	0	8.632
04.08.2011	23:00:00	50	0	0	0	0	0	0	7.882
04.08.2011	00:00:00	50	0	0	0	0	0	0	7.659
04.08.2011	01:00:00	50	0	0	0	0	0	0	8.606
04.08.2011	02:00:00	50	0	0	0	0	0	0	6.494
04.08.2011	03:00:00	50	0	0	0	0	0	0	6.603
04.08.2011	04:00:00	50	1	0.5	0	0.5	0	0	1.729
04.08.2011	05:00:00	50	1	0.5	0.5	0	0	0	1.18
07.08.2011	22:00:00	50	0	0	0	0	0	0	3.34
07.08.2011	23:00:00	50	0	0	0	0	0	0	1.526
07.08.2011	00:00:00	50	0	0	0	0	0	0	1.084
07.08.2011	01:00:00	50	0	0	0	0	0	0	1.837
07.08.2011	02:00:00	50	0	0	0	0	0	0	1.618
07.08.2011	03:00:00	50	0	0	0	0	0	0	1.1
07.08.2011	04:00:00	50	0	0	0	0	0	0	0.699
07.08.2011	05:00:00	50	0	0	0	0	0	0	1.345
19.08.2011	22:00:00	50	12	6	4.5	1	0	0	1.948
19.08.2011	23:00:00	50	0	0	0	0	0	0	0.996
19.08.2011	00:00:00	50	0	0	0	0	0	0	1.302
19.08.2011	01:00:00	50	0	0	0	0	0	0	2.152
19.08.2011	02:00:00	50	0	0	0	0	0	0	3.066
19.08.2011	03:00:00	50	0	0	0	0	0	0	3.004
19.08.2011	04:00:00	50	0	0	0	0	0	0	1.943
19.08.2011	05:00:00	50	0	0	0	0	0	0	1.864
20.08.2011	22:00:00	50	32	16	2.5	2	0	0	2.07
20.08.2011	23:00:00	50	2	1	0.5	0.5	0	0	0.962
20.08.2011	00:00:00	50	0	0	0	0	0	0	1.875
20.08.2011	01:00:00	50	0	0	0	0	0	0	3.031
20.08.2011	02:00:00	50	0	0	0	0	0	0	2.777
20.08.2011	03:00:00	50	0	0	0	0	0	0	2.913
20.08.2011	04:00:00	50	1	0.5	0.5	0	0	0	3.123
20.08.2011	05:00:00	50	1	0.5	0	0	0	0	3.574
21.08.2011	22:00:00	50	5	2.5	1	1.5	0	0	2.012
21.08.2011	23:00:00	50	2	1	0	0	0	1	2.716
21.08.2011	00:00:00	50	0	0	0	0	0	0	3.087
21.08.2011	01:00:00	50	0	0	0	0	0	0	2.657
21.08.2011	02:00:00	50	1	0.5	0	0	0	0.5	1.708
21.08.2011	03:00:00	50	0	0	0	0	0	0	1.506
21.08.2011	04:00:00	50	0	0	0	0	0	0	2.087
21.08.2011	05:00:00	50	1	0.5	0.5	0	0	0	2.985
30.09.2011	21:00:00	50	3	1.5	1.5	0	0	0	1.145
30.09.2011	22:00:00	50	1	0.5	0	0	0	0	0.819
30.09.2011	23:00:00	50	0	0	0	0	0	0	0.633
30.09.2011	00:00:00	50	0	0	0	0	0	0	1.551
30.09.2011	01:00:00	50	0	0	0	0	0	0	2.563
30.09.2011	02:00:00	50	1	0.5	0.5	0	0	0	1.822
30.09.2011	03:00:00	50	0	0	0	0	0	0	1.137
30.09.2011	04:00:00	50	0	0	0	0	0	0	1.655
30.09.2011	05:00:00	50	0	0	0	0	0	0	2.394
30.09.2011	06:00:00	50	0	0	0	0	0	0	3.02
30.09.2011	07:00:00	50	0	0	0	0	0	0	3.129
02.10.2011	21:00:00	50	0	0	0	0	0	0	3.825
02.10.2011	22:00:00	50	0	0	0	0	0	0	0.953
02.10.2011	23:00:00	50	0	0	0	0	0	0	1.773
02.10.2011	00:00:00	50	0	0	0	0	0	0	0.819
02.10.2011	01:00:00	50	0	0	0	0	0	0	1.862
02.10.2011	02:00:00	50	0	0	0	0	0	0	2.559
02.10.2011	03:00:00	50	0	0	0	0	0	0	3.585
02.10.2011	04:00:00	50	0	0	0	0	0	0	2.683
02.10.2011	05:00:00	50	0	0	0	0	0	0	1.949
02.10.2011	06:00:00	50	0	0	0	0	0	0	1.762
02.10.2011	07:00:00	50	0	0	0	0	0	0	2.399

S3 Table continued

15.07.2011	01:00:00	65	1	0.5	0.5	0	0	0	2.489
15.07.2011	02:00:00	65	6	3	2	0.5	0	0	1.766
15.07.2011	03:00:00	65	10	5	4.5	0.5	0	0	1.041
15.07.2011	04:00:00	65	17	8.5	8.5	0	0	0	1.226
15.07.2011	05:00:00	65	11	5.5	3.5	0	0	2	0.911
16.07.2011	22:00:00	65	6	3	0	0.5	0	0	5.202
16.07.2011	23:00:00	65	0	0	0	0	0	0	4.717
16.07.2011	00:00:00	65	0	0	0	0	0	0	5.013
16.07.2011	01:00:00	65	0	0	0	0	0	0	6.920
16.07.2011	02:00:00	65	0	0	0	0	0	0	5.451
16.07.2011	03:00:00	65	1	0.5	0	0	0	0	6.422
16.07.2011	04:00:00	65	0	0	0	0	0	0	3.841
16.07.2011	05:00:00	65	2	1	0	0.5	0	0.5	4.056
04.08.2011	22:00:00	65	1	0.5	0	0.5	0	0	9.023
04.08.2011	23:00:00	65	1	0.5	0	0	0.5	0	8.273
04.08.2011	00:00:00	65	0	0	0	0	0	0	8.016
04.08.2011	01:00:00	65	0	0	0	0	0	0	9.055
04.08.2011	02:00:00	65	0	0	0	0	0	0	6.914
04.08.2011	03:00:00	65	1	0.5	0.5	0	0	0	7.030
04.08.2011	04:00:00	65	4	2	0	2	0	0	1.837
04.08.2011	05:00:00	65	2	1	0.5	0.5	0	0	1.196
07.08.2011	22:00:00	65	7	3.5	0	0.5	0	0	3.645
07.08.2011	23:00:00	65	2	1	0	0	0	1	1.609
07.08.2011	00:00:00	65	1	0.5	0.5	0	0	0	1.122
07.08.2011	01:00:00	65	0	0	0	0	0	0	1.886
07.08.2011	02:00:00	65	14	7	6.5	0	0	0.5	1.760
07.08.2011	03:00:00	65	0	0	0	0	0	0	1.152
07.08.2011	04:00:00	65	0	0	0	0	0	0	0.661
07.08.2011	05:00:00	65	5	2.5	0	0	0	2.5	1.363
19.08.2011	22:00:00	65	30	15	7	5.5	0	0	2.092
19.08.2011	23:00:00	65	6	3	2.5	0.5	0	0	1.025
19.08.2011	00:00:00	65	4	2	1	0	0	0.5	1.392
19.08.2011	01:00:00	65	2	1	0.5	0	0	0	2.332
19.08.2011	02:00:00	65	0	0	0	0	0	0	3.327
19.08.2011	03:00:00	65	0	0	0	0	0	0	3.235
19.08.2011	04:00:00	65	1	0.5	0	0	0	0	2.143
19.08.2011	05:00:00	65	0	0	0	0	0	0	2.035
20.08.2011	22:00:00	65	10	5	1.5	2.5	0	0	2.410
20.08.2011	23:00:00	65	5	2.5	1	1	0	0	0.953
20.08.2011	00:00:00	65	2	1	0	1	0	0	2.050
20.08.2011	01:00:00	65	2	1	1	0	0	0	3.305
20.08.2011	02:00:00	65	2	1	0	1	0	0	3.080
20.08.2011	03:00:00	65	3	1.5	0	0	0	0.5	3.228
20.08.2011	04:00:00	65	1	0.5	0.5	0	0	0	3.507
20.08.2011	05:00:00	65	7	3.5	0	0	0	0	4.010
21.08.2011	22:00:00	65	10	5	1	4	0	0	2.133
21.08.2011	23:00:00	65	9	4.5	0	0	0	4.5	2.885
21.08.2011	00:00:00	65	0	0	0	0	0	0	3.357
21.08.2011	01:00:00	65	3	1.5	1	0	0	0	2.911
21.08.2011	02:00:00	65	4	2	0	0	0	2	1.883
21.08.2011	03:00:00	65	4	2	0	0	0	2	1.632
21.08.2011	04:00:00	65	1	0.5	0.5	0	0	0	2.291
21.08.2011	05:00:00	65	0	0	0	0	0	0	3.309
30.09.2011	21:00:00	65	6	3	1.5	1.5	0	0	1.163
30.09.2011	22:00:00	65	2	1	1	0	0	0	0.868
30.09.2011	23:00:00	65	0	0	0	0	0	0	0.660
30.09.2011	00:00:00	65	1	0.5	0.5	0	0	0	1.693
30.09.2011	01:00:00	65	0	0	0	0	0	0	2.864
30.09.2011	02:00:00	65	0	0	0	0	0	0	2.013
30.09.2011	03:00:00	65	0	0	0	0	0	0	1.238
30.09.2011	04:00:00	65	0	0	0	0	0	0	1.808
30.09.2011	05:00:00	65	0	0	0	0	0	0	2.646
30.09.2011	06:00:00	65	0	0	0	0	0	0	3.368
30.09.2011	07:00:00	65	0	0	0	0	0	0	3.471
02.10.2011	21:00:00	65	0	0	0	0	0	0	4.169
02.10.2011	22:00:00	65	0	0	0	0	0	0	0.937
02.10.2011	23:00:00	65	0	0	0	0	0	0	1.910
02.10.2011	00:00:00	65	0	0	0	0	0	0	0.895
02.10.2011	01:00:00	65	0	0	0	0	0	0	2.073
02.10.2011	02:00:00	65	0	0	0	0	0	0	2.865
02.10.2011	03:00:00	65	0	0	0	0	0	0	4.012
02.10.2011	04:00:00	65	0	0	0	0	0	0	2.995
02.10.2011	05:00:00	65	0	0	0	0	0	0	2.210
02.10.2011	06:00:00	65	0	0	0	0	0	0	1.987
02.10.2011	07:00:00	65	0	0	0	0	0	0	2.711
