# Supplementary material for: Mitigating the negative impacts of tall wind turbines on bats: Vertical activity profiles and relationships to wind speed
Source: PLoS One. 2018 Mar 21;13(3):e0192493. doi: 10.1371/journal.pone.0192493 (PMC5862399; doi:10.1371/journal.pone.0192493)
Supplement: S5 Table — (PDF) [file pone.0192493.s007.pdf]

Supporting information

S5 Table. Overview of hourly number of bat passes and wind speed at the six projected wind turbine sites.

Date	Time	Total bat passes	Bat passes Plantation	Bat passes Open field	Bat passes *P. pipistrellus*	Bat passes *M. myotis/M. blythii*	Bat passes *T. teniotis*	V35 [m/s]	Extrapolated V5 [m/s]
12.07.2011	22:00:00	1	1	0	1	0	0	1.950	1.545
12.07.2011	23:00:00	65	28	37	39	4	0	4.950	3.921
12.07.2011	00:00:00	55	30	25	30	1	0	5.533	4.384
12.07.2011	01:00:00	29	17	12	17	6	0	7.233	5.730
12.07.2011	02:00:00	17	8	9	8	1	0	6.933	5.493
12.07.2011	03:00:00	30	10	20	12	0	15	8.367	6.628
12.07.2011	04:00:00	3	1	2	0	2	0	3.017	2.390
12.07.2011	05:00:00	8	3	5	4	0	0	3.200	2.535
18.07.2011	22:00:00	3	0	3	3	0	0	6.950	5.506
18.07.2011	23:00:00	120	16	104	111	0	0	5.983	4.740
18.07.2011	00:00:00	11	3	8	6	1	2	2.400	1.901
18.07.2011	01:00:00	24	4	20	7	5	7	1.283	1.017
18.07.2011	02:00:00	7	1	6	4	2	0	1.033	0.819
18.07.2011	03:00:00	9	3	6	4	0	5	0.767	0.607
18.07.2011	04:00:00	6	0	6	0	0	6	0.883	0.700
18.07.2011	05:00:00	2	0	2	0	0	2	1.250	0.990
21.07.2011	22:00:00	67	2	65	60	0	0	6.083	4.819
21.07.2011	23:00:00	75	0	75	70	2	0	4.783	3.789
21.07.2011	00:00:00	11	0	11	10	0	1	3.033	2.403
21.07.2011	01:00:00	29	4	25	26	0	3	2.033	1.611
21.07.2011	02:00:00	7	2	5	5	0	0	1.750	1.386
21.07.2011	03:00:00	14	0	14	9	0	5	1.333	1.056
21.07.2011	04:00:00	4	0	4	2	1	0	1.667	1.320
21.07.2011	05:00:00	2	2	0	2	0	0	1.767	1.400
01.08.2011	22:00:00	3	2	1	2	0	0	7.067	5.598
01.08.2011	23:00:00	23	4	19	10	10	0	6.833	5.413
01.08.2011	00:00:00	45	2	43	30	7	0	2.267	1.796
01.08.2011	01:00:00	18	1	17	11	1	0	1.567	1.241
01.08.2011	02:00:00	10	2	8	8	5	0	0.183	0.145
01.08.2011	03:00:00	18	5	13	17	0	0	0.500	0.396
01.08.2011	04:00:00	8	2	6	7	0	0	0.917	0.726
01.08.2011	05:00:00	2	0	2	2	0	0	0.900	0.713
10.08.2011	22:00:00	199	4	195	178	1	0	7.583	6.008
10.08.2011	23:00:00	25	1	24	24	0	0	3.583	2.839
10.08.2011	00:00:00	34	5	29	34	0	0	1.867	1.479
10.08.2011	01:00:00	71	8	63	68	1	2	0.767	0.607
10.08.2011	02:00:00	9	3	6	8	0	0	0.683	0.541
10.08.2011	03:00:00	2	2	0	2	0	0	1.133	0.898
10.08.2011	04:00:00	1	0	1	0	1	0	0.600	0.475
10.08.2011	05:00:00	0	0	0	0	0	0	1.217	0.964
11.08.2011	22:00:00	53	5	48	24	0	0	7.500	5.942
11.08.2011	23:00:00	21	2	19	20	0	0	7.600	6.021
11.08.2011	00:00:00	28	2	26	27	0	0	7.017	5.559
11.08.2011	01:00:00	19	3	16	18	0	0	2.583	2.047
11.08.2011	02:00:00	24	8	16	23	1	0	1.600	1.268
11.08.2011	03:00:00	50	12	38	49	2	0	1.283	1.017
11.08.2011	04:00:00	4	0	4	3	0	0	0.850	0.673
11.08.2011	05:00:00	3	1	2	3	0	0	0.767	0.607
28.08.2011	22:00:00	106	16	90	93	4	0	1.583	1.254
28.08.2011	23:00:00	126	15	111	116	6	0	1.417	1.122
28.08.2011	00:00:00	36	10	26	29	1	0	1.100	0.871
28.08.2011	01:00:00	27	16	11	16	0	0	1.250	0.990
28.08.2011	02:00:00	7	3	4	1	2	0	0.467	0.370
28.08.2011	03:00:00	7	2	5	0	0	5	0.933	0.739
28.08.2011	04:00:00	3	3	0	0	0	0	0.667	0.528
28.08.2011	05:00:00	9	2	7	0	0	8	0.450	0.356
01.09.2011	22:00:00	127	8	119	114	1	0	1.617	1.281
01.09.2011	23:00:00	84	15	69	64	7	0	1.333	1.056
01.09.2011	00:00:00	21	6	15	13	1	0	5.700	4.516
01.09.2011	01:00:00	21	9	12	9	1	0	4.933	3.908
01.09.2011	02:00:00	15	4	11	8	3	0	4.400	3.486
01.09.2011	03:00:00	22	11	11	6	3	0	4.700	3.723
01.09.2011	04:00:00	9	0	9	2	6	0	4.150	3.288
01.09.2011	05:00:00	22	4	18	13	7	0	4.333	3.433

S4 Table continued

06.09.2011	21:00:00	196	5	191	186	1	0	2.150	1.703
06.09.2011	22:00:00	78	23	55	73	0	0	1.083	0.858
06.09.2011	23:00:00	54	15	39	49	0	0	1.967	1.558
06.09.2011	00:00:00	9	3	6	5	0	3	0.733	0.581
06.09.2011	01:00:00	5	1	4	5	0	0	0.967	0.766
06.09.2011	02:00:00	1	1	0	0	0	0	1.433	1.135
06.09.2011	03:00:00	0	0	0	0	0	0	1.233	0.977
06.09.2011	04:00:00	2	0	2	0	0	2	0.850	0.673
06.09.2011	05:00:00	0	0	0	0	0	0	1.517	1.202
06.09.2011	06:00:00	0	0	0	0	0	0	1.850	1.466
14.10.2011	20:00:00	1	0	1	1	0	0	2.833	2.245
14.10.2011	21:00:00	3	1	2	2	0	0	1.383	1.096
14.10.2011	22:00:00	0	0	0	0	0	0	0.367	0.290
14.10.2011	23:00:00	0	0	0	0	0	0	1.200	0.951
14.10.2011	00:00:00	0	0	0	0	0	0	0.433	0.343
14.10.2011	01:00:00	0	0	0	0	0	0	0.717	0.568
14.10.2011	02:00:00	0	0	0	0	0	0	1.017	0.805
14.10.2011	03:00:00	0	0	0	0	0	0	0.900	0.713
14.10.2011	04:00:00	0	0	0	0	0	0	1.417	1.122
14.10.2011	05:00:00	0	0	0	0	0	0	0.900	0.713
14.10.2011	06:00:00	0	0	0	0	0	0	1.000	0.792
14.10.2011	07:00:00	0	0	0	0	0	0	1.467	1.162
14.10.2011	08:00:00	0	0	0	0	0	0	1.750	1.386
15.10.2011	20:00:00	0	0	0	0	0	0	0.733	0.581
15.10.2011	21:00:00	0	0	0	0	0	0	0.400	0.317
15.10.2011	22:00:00	0	0	0	0	0	0	0.717	0.568
15.10.2011	23:00:00	0	0	0	0	0	0	0.650	0.515
15.10.2011	00:00:00	0	0	0	0	0	0	0.967	0.766
15.10.2011	01:00:00	0	0	0	0	0	0	1.517	1.202
15.10.2011	02:00:00	0	0	0	0	0	0	1.800	1.426
15.10.2011	03:00:00	0	0	0	0	0	0	1.483	1.175
15.10.2011	04:00:00	0	0	0	0	0	0	1.433	1.135
15.10.2011	05:00:00	0	0	0	0	0	0	1.267	1.003
15.10.2011	06:00:00	0	0	0	0	0	0	1.233	0.977
15.10.2011	07:00:00	0	0	0	0	0	0	1.283	1.017
15.10.2011	08:00:00	0	0	0	0	0	0	0.800	0.634
03.05.2012	21:00:00	0	0	0	0	0	0	4.833	3.829
03.05.2012	22:00:00	11	1	10	10	0	0	3.433	2.720
03.05.2012	23:00:00	1	0	1	1	0	0	2.667	2.113
03.05.2012	00:00:00	2	1	1	1	0	0	1.367	1.083
03.05.2012	01:00:00	1	0	1	1	0	0	1.317	1.043
03.05.2012	02:00:00	0	0	0	0	0	0	0.533	0.422
03.05.2012	03:00:00	0	0	0	0	0	0	0.833	0.660
03.05.2012	04:00:00	0	0	0	0	0	0	1.000	0.792
03.05.2012	05:00:00	0	0	0	0	0	0	1.167	0.925
07.05.2012	22:00:00	20	0	20	18	0	0	7.183	5.690
07.05.2012	23:00:00	9	0	9	9	0	0	5.467	4.331
07.05.2012	00:00:00	0	0	0	0	0	0	3.000	2.377
07.05.2012	01:00:00	2	1	1	2	0	0	1.367	1.083
07.05.2012	02:00:00	2	0	2	2	0	0	1.550	1.228
07.05.2012	03:00:00	0	0	0	0	0	0	1.000	0.792
07.05.2012	04:00:00	0	0	0	0	0	0	0.867	0.687
07.05.2012	05:00:00	0	0	0	0	0	0	0.950	0.753
16.05.2012	22:00:00	0	0	0	0	0	0	5.017	3.974
16.05.2012	23:00:00	0	0	0	0	0	0	0.817	0.647
16.05.2012	00:00:00	0	0	0	0	0	0	1.317	1.043
16.05.2012	01:00:00	0	0	0	0	0	0	1.217	0.964
16.05.2012	02:00:00	0	0	0	0	0	0	1.250	0.990
16.05.2012	03:00:00	0	0	0	0	0	0	0.300	0.238
16.05.2012	04:00:00	0	0	0	0	0	0	0.933	0.739
16.05.2012	05:00:00	0	0	0	0	0	0	0.600	0.475

S4 Table continued

24.05.2012	22:00:00	0	0	0	0	0	0	2.633	2.086
24.05.2012	23:00:00	2	1	1	2	0	0	1.317	1.043
24.05.2012	00:00:00	1	0	1	1	0	0	2.133	1.690
24.05.2012	01:00:00	5	1	4	5	0	0	0.600	0.475
24.05.2012	02:00:00	1	0	1	1	0	0	0.600	0.475
24.05.2012	03:00:00	1	0	1	1	0	0	0.667	0.528
24.05.2012	04:00:00	0	0	0	0	0	0	1.000	0.792
24.05.2012	05:00:00	0	0	0	0	0	0	0.600	0.475
13.06.2012	22:00:00	0	0	0	0	0	0	7.250	5.743
13.06.2012	23:00:00	1	0	1	0	0	0	6.700	5.308
13.06.2012	00:00:00	0	0	0	0	0	0	4.667	3.697
13.06.2012	01:00:00	0	0	0	0	0	0	2.033	1.611
13.06.2012	02:00:00	1	1	0	1	0	0	0.750	0.594
13.06.2012	03:00:00	0	0	0	0	0	0	0.683	0.541
13.06.2012	04:00:00	0	0	0	0	0	0	0.900	0.713
17.06.2012	22:00:00	32	0	32	32	0	0	6.467	5.123
17.06.2012	23:00:00	19	0	19	15	0	3	2.067	1.637
17.06.2012	00:00:00	1	0	1	1	0	0	1.383	1.096
17.06.2012	01:00:00	0	0	0	0	0	0	0.567	0.449
17.06.2012	02:00:00	4	0	4	3	0	0	1.233	0.977
17.06.2012	03:00:00	1	0	1	1	0	0	1.700	1.347
17.06.2012	04:00:00	3	0	3	1	1	0	1.467	1.162
26.06.2012	22:00:00	42	0	42	42	0	0	8.883	7.037
26.06.2012	23:00:00	59	0	59	59	0	0	6.783	5.374
26.06.2012	00:00:00	35	0	35	35	0	0	3.967	3.143
26.06.2012	01:00:00	7	0	7	6	0	0	1.083	0.858
26.06.2012	02:00:00	2	0	2	1	0	0	0.717	0.568
26.06.2012	03:00:00	0	0	0	0	0	0	0.800	0.634
26.06.2012	04:00:00	0	0	0	0	0	0	0.483	0.383
29.06.2012	22:00:00	3	0	3	1	0	0	1.933	1.531
29.06.2012	23:00:00	13	0	13	13	0	0	1.467	1.162
29.06.2012	00:00:00	9	1	8	8	0	0	0.967	0.766
29.06.2012	01:00:00	0	0	0	0	0	0	1.000	0.792
29.06.2012	02:00:00	0	0	0	0	0	0	1.183	0.937
29.06.2012	03:00:00	0	0	0	0	0	0	1.633	1.294
29.06.2012	04:00:00	1	0	1	0	0	0	1.467	1.162
