# Supplementary figures and images for: Mitigating the negative impacts of tall wind turbines on bats: Vertical activity profiles and relationships to wind speed
Source: PLoS One. 2018 Mar 21;13(3):e0192493. doi: 10.1371/journal.pone.0192493 (PMC5862399; doi:10.1371/journal.pone.0192493)

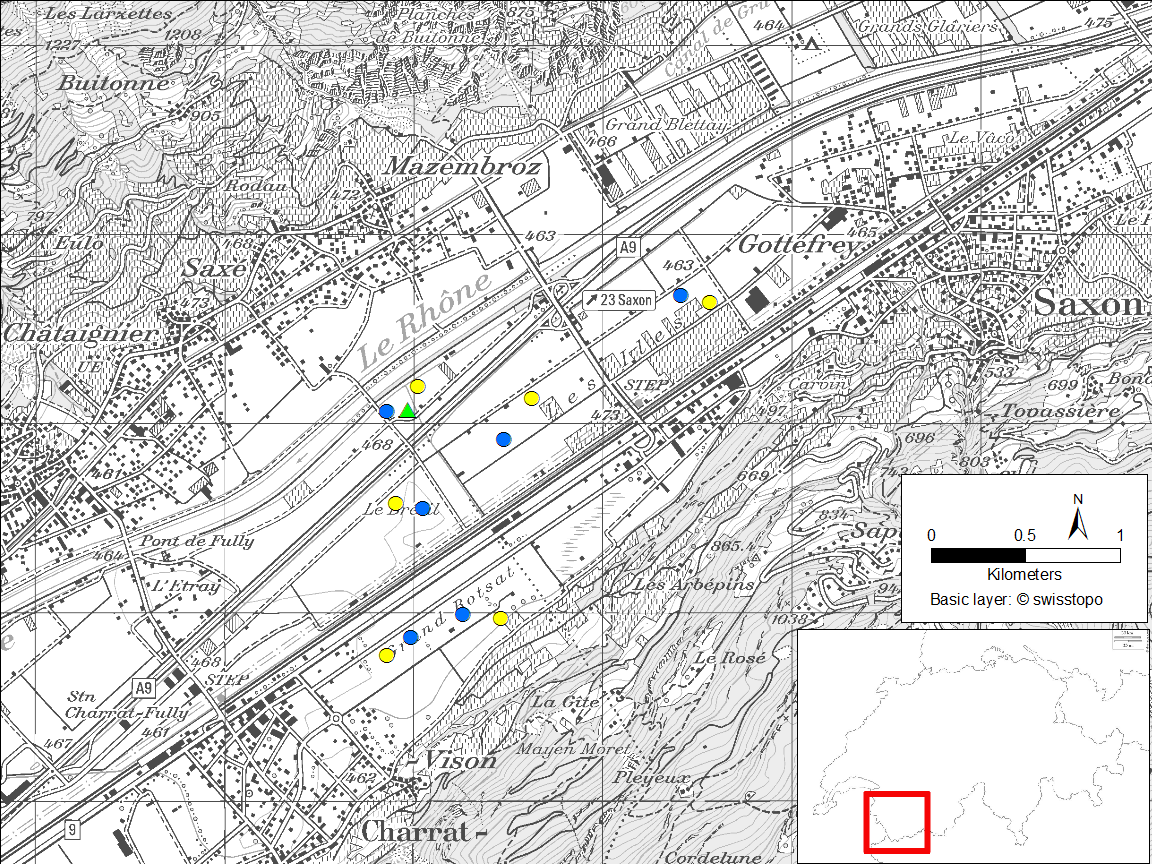

Supplement: S1 Fig — Round symbols indicate recordings at foreseen wind turbine sites (ValEole 1–6; yellow: fruit tree plantations, blue: open fields). The green triangle indicates the first site for vertical recordings (Solverse). The second site (Marais d’Ardon) is situated 10 km northeast and therefore not depicted here. (TIFF) [file pone.0192493.s008.tiff]

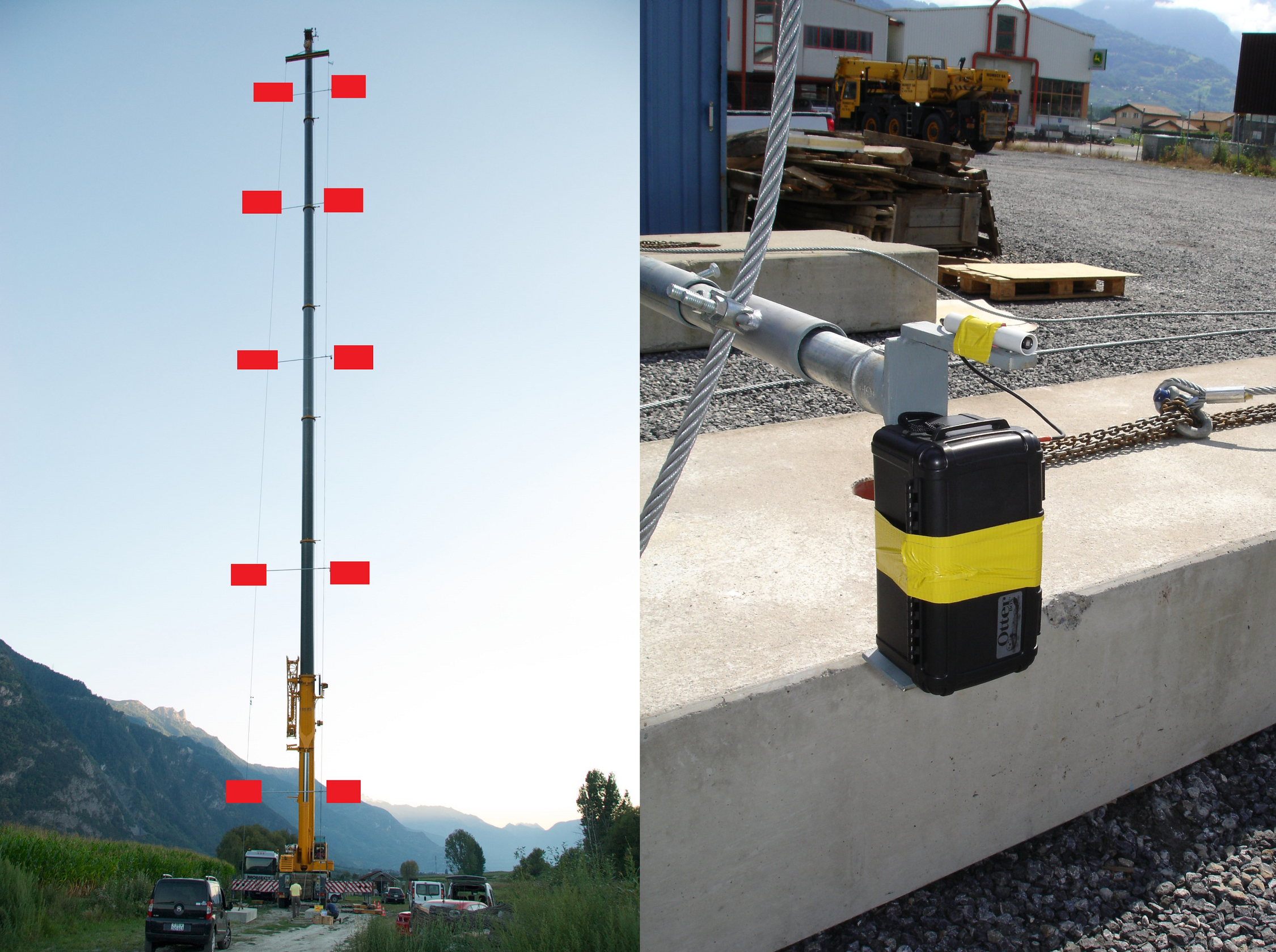

Supplement: S2 Fig — Left: Installation of the bat recorders on the truck-mounted crane (vertical activity profiles): two 2 cm thick metal cables were stretched from the ground level (fixed on two cement blocks of 2.5 tons each) to a 4 m long horizontal bar fixed under the crane hook, which was pulled up to 70 m above ground. Thereby the cables were kept under permanent high tension in order to avoid to whole system to twist. The distance of 4 m between the two vertical cables was maintained by horizontal metal bars (4.5 m each, positioned at 5 m, 20 m, 35 m, 50 m and 65 m a.g.l., respectively) at both ends of which the bat detectors (red squares) were fixed. Two additional recorders were attached to the uppermost bar at 70 m a.g.l., resulting in 12 recorders in total. Right: bat detector (inside the black protection box) attached at one end of a horizontal metal bar. The microphone is inside a plastic protection tube (white). During measurements microphones were directed downwards with an angle of approximately 30° to prevent damage from rain. (TIFF) [file pone.0192493.s009.tiff]

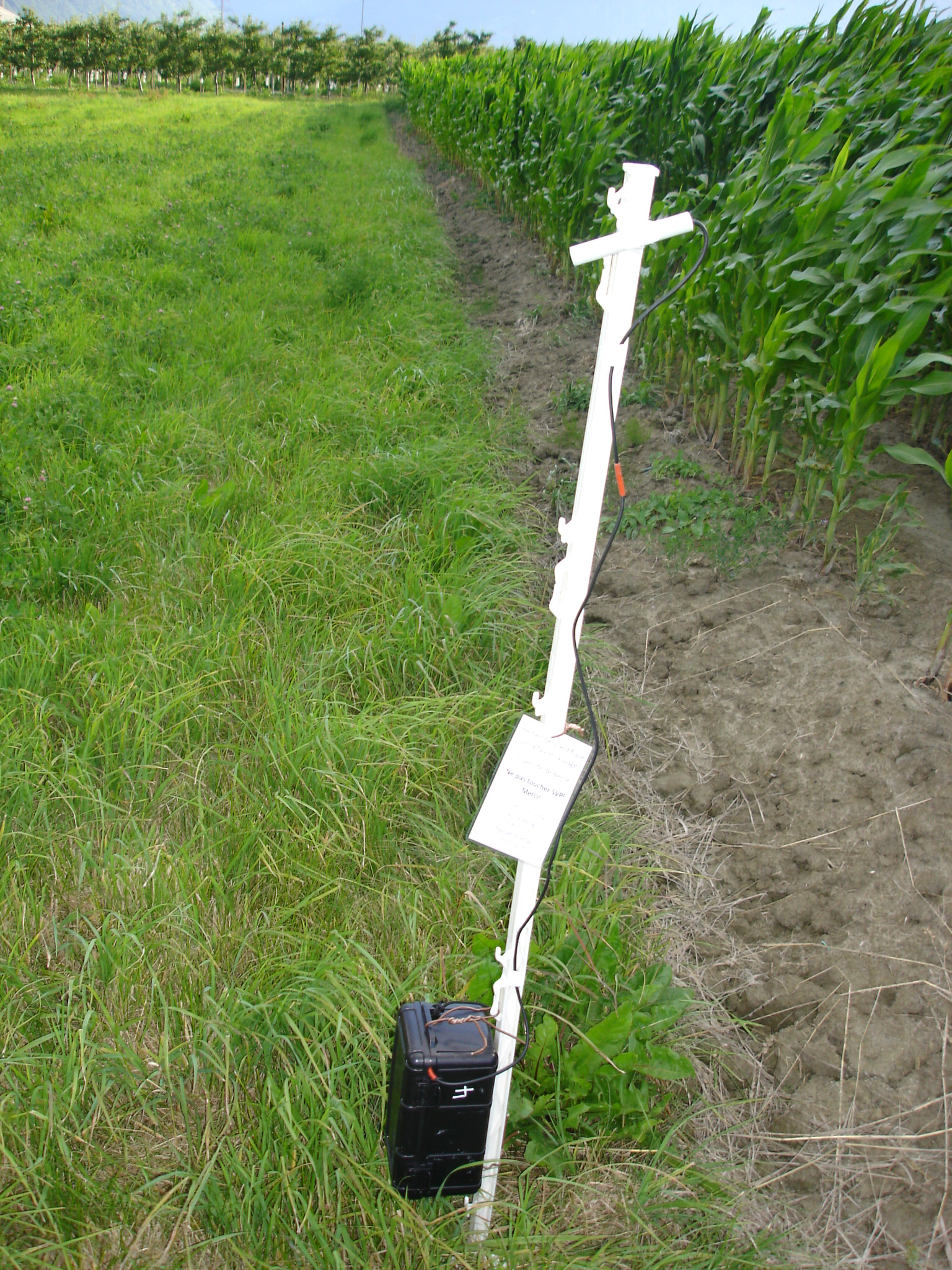

Supplement: S3 Fig — The microphone is inside a plastic protection tube (white) and the bat detector is inside the protection box (black). Microphones were directed downwards with an angle of approximately 30° to prevent damage from rain. (TIFF) [file pone.0192493.s010.tiff]

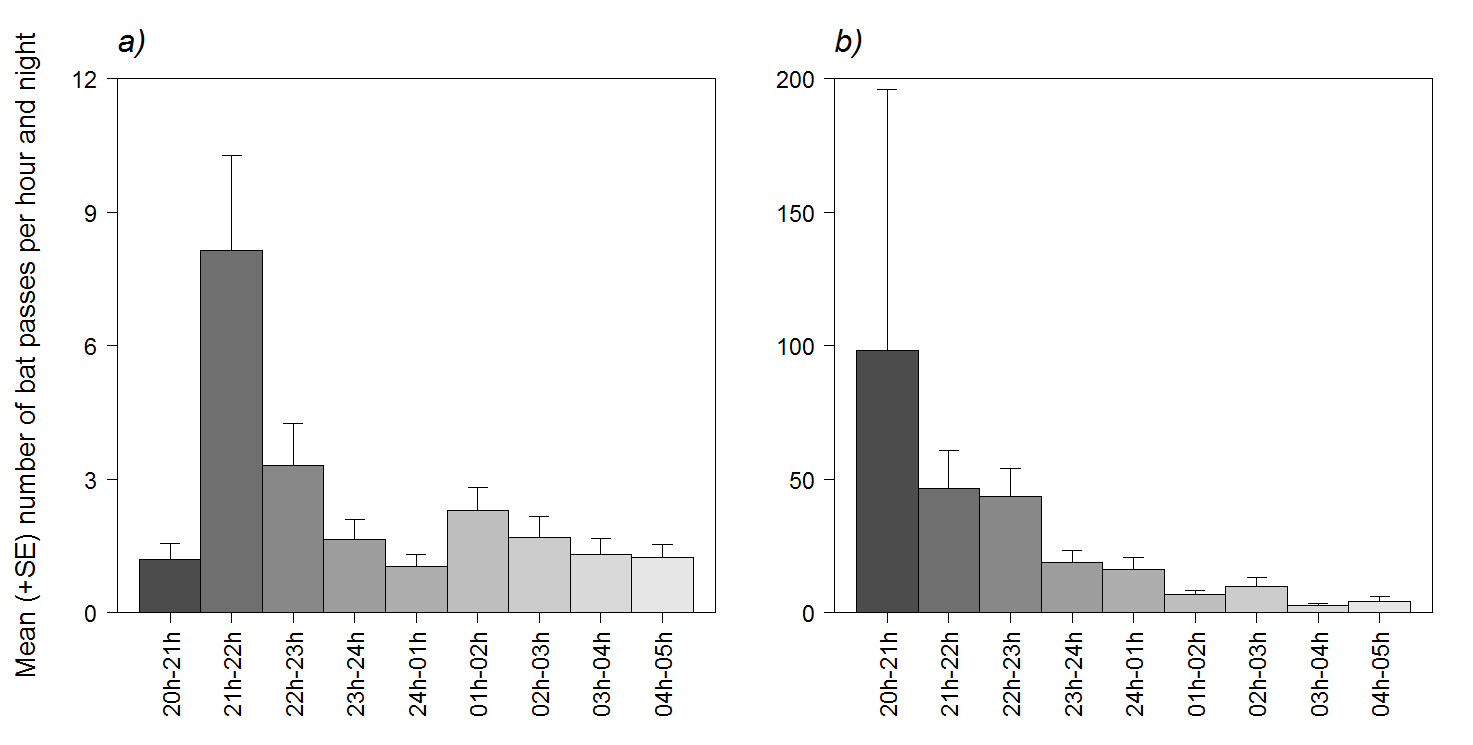

Supplement: S6 Fig — Data are shown for all species pooled, as recorded at a) the truck-mounted crane and b) at ground level at the six foreseen wind turbine sites. Error bars indicate the standard error of the mean (SE). (TIFF) [file pone.0192493.s013.tiff]

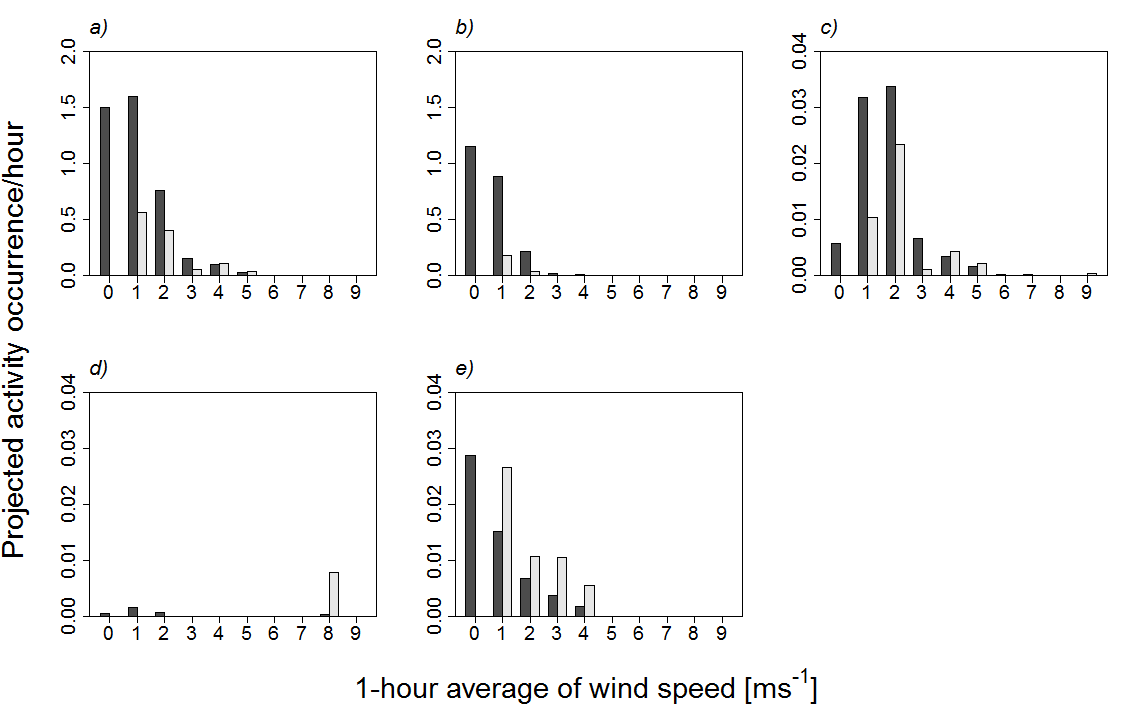

Supplement: S7 Fig — Models were calculated for a) all bat species pooled, b) P. pipistrellus, c) H. savii, d) M. myotis/M. blythii and e) T. teniotis. Black bars: all heights pooled; white bars: only heights ≥ 50 m a.g.l.. (TIFF) [file pone.0192493.s014.tiff]
